# A numerical study of mean corrected phase-averaging in geophysical flow equations

**DOI:** 10.1007/s00162-026-00797-x

**Published:** 2026-09-03

**Authors:** Timothy C. Andrews, Beth A. Wingate

**Affiliations:** 1 Department of Mathematics and Statistics, University of Exeter, Exeter, UK; 2 Department of Climate and Space Sciences and Engineering, University of Michigan, Ann Arbor, USA

**Keywords:** Phase-averaging, Timestepping, Geophysical flows, Shallow water equations

## Abstract

**Abstract:**

This paper introduces a new algorithm to improve the accuracy of numerical phase-averaging in the oscillatory, multiscale, differential equations that govern geophysical fluid flow. Phase-averaging is a timestepping method that averages a mapped modulation variable in which the equations no longer contain a highly oscillatory linear term. This allows phase-averaging to retain the main contribution of fast waves on the low frequencies without explicitly resolving the rapid oscillations, thus allowing larger timesteps for explicit timesteppers. Whilst the modulation variable mapping is also used in operator splitting methods, and is accurate for asymptotic regimes, the advantage of phase-averaging is that low-frequency oscillations are retained, allowing for accuracy in systems further from the asymptotic limit. However, this comes at the cost of introducing an averaging error when computing phase-averages using a smooth kernel. This paper proposes a method to offset some averaging error through a modified mapping that includes a mean correction term to capture an average measure of nonlinear oscillation. We show that the mean corrected algorithm reduces phase-averaging errors in the swinging spring system and the one-dimensional rotating shallow water equations, which are an important geophysical fluid system for weather and climate applications. We discuss potential new directions for the method based on the outcome of the numerical experiments.

**Graphical abstract:**

**Figure:**
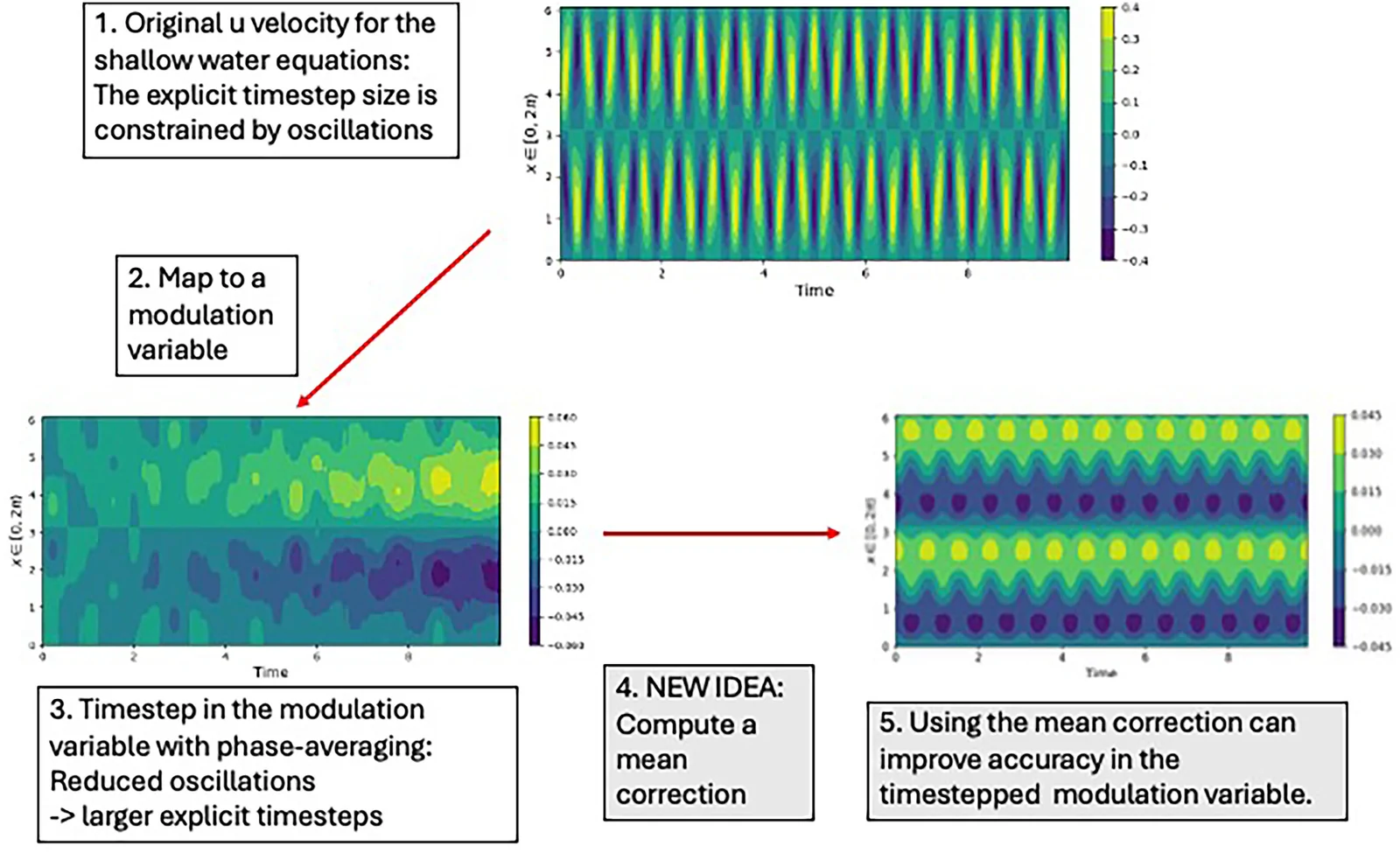

## Introduction

Two challenges to numerically solving the equations that govern atmospheric fluid flow are (1) the presence of multiple time scales and (2) numerical stiffness from fast oscillations. For explicit timestepping methods, accuracy and stability restrictions can then cause timesteps to be prohibitively small. This is undesirable in weather and climate models, as the necessity of generating operational forecasts in a timely manner makes the reduction of wallclock time a key objective [1]. In past decades, this motivated research into new timestepping methods, such as semi-implicit semi-Lagrangian [2–4], implicit-explicit (IMEX) [5–7] and multiscale [8–12] schemes. These types of timestepping methods enabled larger timesteps or shorter time-to-solution for global weather forecasting models without sacrificing accuracy [13].

Some multiscale methods, such as the operator splitting method of [8], are based on exact asymptotic representations of limiting solutions proposed by numerous studies [14–18]. The idea is to map to a *modulation variable* space that is less oscillatory and more conducive for explicit timestepping. Classical averaging methods, which remove secular terms, are then used to filter out the oscillatory waves and leave a system that is asymptotically exact [16, 17, 19]. This approach is accurate and computationally cheap for regimes with a sufficiently large separation of timescales. However, this type of operator splitting loses accuracy when the fluid flow is further from the asymptotic limit of rapid rotation, which restricts its use for more general geophysical flows.

To enable a modulation variable approach to be effective outside the asymptotic limit, a modified method called *phase-averaging* was proposed by [20]. Phase-averaging retains the mapping to a modulation variable, but replaces the classical average with a local time average over the phase. The phase-average is computed numerically using a smooth kernel with a compact support; this finite averaging interval permits slowly oscillating waves in the averaged system and allows for accuracy away from the asymptotic limit. In geophysical fluid systems, the low frequency components can be crucial for dynamics over long time periods, e.g. [21–23].

The error of the phase-averaging method was shown by [20] to be a function of the timestep size, ΔT, the averaging window length, η, and a parameter ϵ, which represents the speed of the fast linear oscillations. [20] also provided an error bound, which provides the intuition that larger averaging windows allow for larger explicit timesteps, but at the cost of a greater averaging error. Advantageously, the error bound for phase-averaging holds not only in the limit of ϵ small, as per classically averaged methods like [8], but also for moderate values of ϵ, including $\epsilon \sim \mathcal {O}(1)$. Phase-averaging has been successfully applied to the rotating shallow water equations (RSWEs) on the *f*-plane [20] and on a spherical domain [24].

Several investigators have proposed methods to reduce the averaging error in the modulation variable, as estimated in [20]. These include higher-order phase-averaging [25], parareal corrections with multiple levels of averaging window [26], and a more general method for correcting phase errors of highly oscillatory equations for the parareal method [27]. Focusing on problems with weak nonlinearity, where a small value of ϵ multiplies the nonlinear term, [28] proposed a method that alters the modulation variable mapping. The new mapping in [28] includes a mean correction that can reduce phase errors, but requires that ϵ is sufficiently small.

**Fig. 1:**
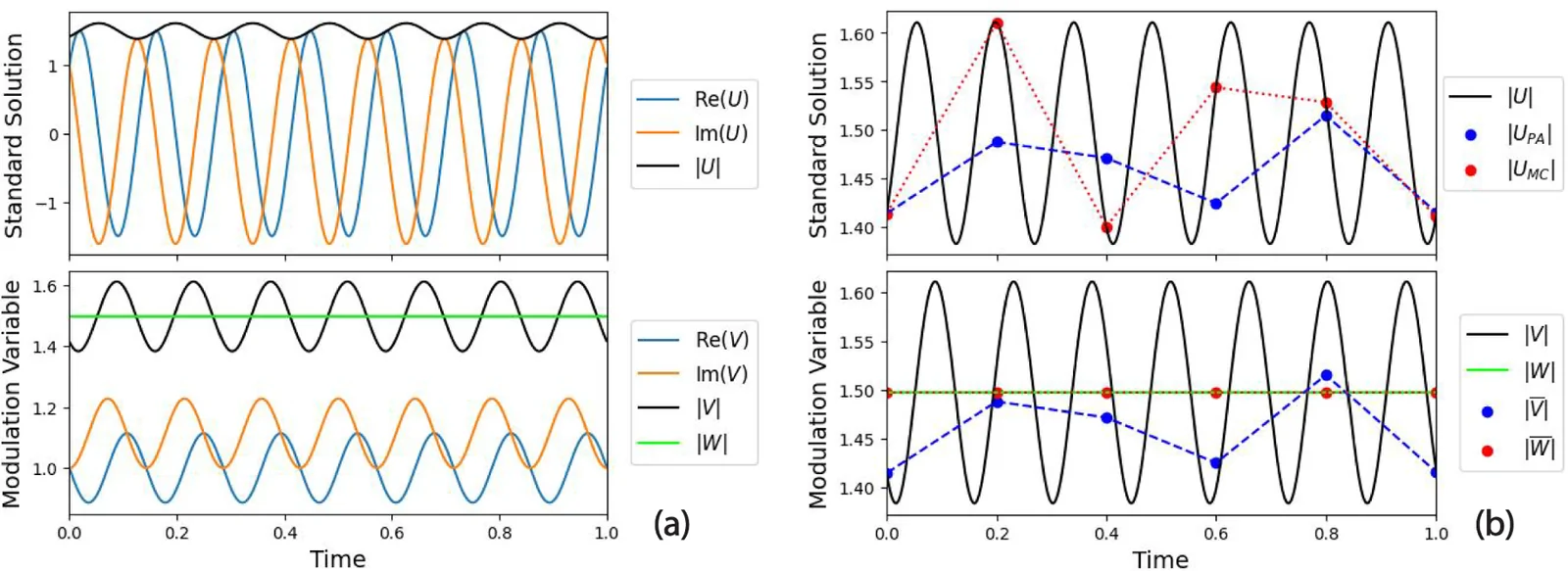
An instance of the simple example ODE (19). The analytical solution *U* is shown in (**a**), along with its modulation variable, *V*, which has a reduced oscillation amplitude in both the real and imaginary parts. Timestepped solutions using the phase-averaged methods are shown in (**b**), computed for $|U(t)| = \sqrt{\text {Re}^2(U) + \text {Im}^2(U)}$, with blue for standard phase-averaging and red for the mean corrected version. With standard phase-averaging, averaging errors in the averaged modulation variable ($\overline{V}$) lead to discrepancies in $|U_{\text {PA}}|$. No averaging error is committed in the mean corrected modulation variable solution, with a constant mean correction in time leading to $|U_{\text {MC}}(t)| = |U(t)|$ in this example. In more complex PDEs, the mean corrected algorithm seeks to exploit such potential accuracy improvements, given the computation of slowly varying (approximately constant) components of nonlinear oscillation

This paper proposes a new method to reduce the averaging error in the phase-averaging method of [20] by adding a mean correction. Our correction is similar to that of [28], but with the distinction that it is computed over a finite time interval to allow accuracy when ϵ is not small. This *local mean correction* averages the nonlinear oscillations of the modulation variable to quantify locally constant-in-time solution components constructed by the nonlinearity. Incorporating the mean correction into the modulation variable mapping avoids these low-frequency components from being subject to averaging error. Intuition as to potential accuracy improvements with the mean correction are described in Sect. 2.3, specifically in Fig. 1 and its caption.

This work will focus on simple geophysical flows, demonstrating the principles of the method numerically, although the new algorithm outlined in Sect. 3 has more general applicability. In two numerical experiments, we explore accuracy improvements with mean corrected phase-averaging compared to standard phase-averaging. The first test uses the swinging spring ordinary differential equation [29]. This system models the resonant nonlinear interactions of three linear waves in the RSWEs, where fast oscillations interact to construct slower nonlinear dynamics. The swinging spring shows the impact of the mean correction through reduced phase errors in the mapped modulation variable space. The second test is with the *f*-plane RSWEs, which [8] used to examine their classically-averaged operator splitting algorithm. The RSWEs are a useful test bed for geophysical fluid applications, e.g. [30–34] and many others, as they are simpler to solve than the full Euler equations that govern atmospheric fluid flow, yet retain the numerical challenges of multiple scales and oscillatory stiffness. We will study a range of linear oscillation speeds, ϵ, to test the mean corrected method both near and far from the asymptotic limit. We experimentally determine the best finite windows to compute the local mean correction, and find that these depend on ϵ.

Following this introduction, Sect. 2 discusses the operator splitting and standard phase-averaging methods, before introducing the classical mean correction of [28] and our new local mean correction. The key differences between our method for multiscale geophysical fluid equations and the previous application in [28] are discussed. Next, Sect. 3 describes the implementation of finite phase-averages and provides pseudocode for the mean corrected phase-averaging algorithm. Our numerical studies are described in Sects. 4 and 5. Finally, Sect. 6 discusses future improvements to the mean corrected algorithm and other applications for this new method.

## Phase-averaging and the mean correction

We firstly describe the phase averaging method and our proposed mean corrected version. An overview of the notation used in this work is provided in Table 1.

**Table 1:** Notation used to describe the standard and mean corrected phase-averaging methods

Variable	Description
$\textbf{U}$	Original variables.
$\textbf{V}$	Modulation variables for the standard phase-averaged method.
$\overline{\textbf{V}}$	Averaged modulation variables for the standard phase-averaged method.
$\textbf{U}_{\text {PA}}$	Original variables from the standard phase-averaged method, obtained by back transforming $\overline{\textbf{V}}$.
$\textbf{W}$	Modulation variables for the mean corrected phase-averaged method.
$\overline{\textbf{W}}$	Averaged modulation variables for the mean corrected phase-averaged method.
$\textbf{U}_{\text {MC}}$	Original variables from the mean corrected phase-averaged method, obtained by back transforming $\overline{\textbf{W}}$.
ϵ	Characteristic linear oscillation speed.
$\mathcal {L}$	Linear operator.
$\mathcal {N}$	Nonlinear operator.
$\textbf{C}$	Mean correction.
$\mathcal {K}(s)$	Averaging kernel for phase-averaging of a timestepping equation, with the averaging performed with respect to the phase variable *s*.
$\mathcal {K}_C(r)$	Averaging kernel for computing a local mean correction, with the averaging performed with respect to the phase variable *r*.
η	Averaging window size for phase-averaging of a timestepping equation.
ζ	Normalised averaging window of $\zeta =\eta /\Delta t$.
$\eta _C$	Averaging window size for computing a local mean correction.

### Phase-averaging

We consider multiscale initial value problems that govern geophysical fluid flow in the form

1 $$\begin{aligned} \frac{\partial \textbf{U}}{\partial t} + \frac{1}{\epsilon } \mathcal {L} \textbf{U} = \mathcal {N}(\textbf{U}), ~\textbf{U}(0)=\textbf{U}_0, ~\epsilon \in (0,1], \end{aligned}$$

where $\textbf{U}(t) = (u_1(t,\cdot ),u_2(t,\cdot ),...)$ is a (spatial) vector-valued function, $\mathcal {L}$ is a skew-symmetric linear operator containing purely imaginary (oscillatory) eigenvalues, and $\mathcal {N}(\textbf{U})$ is an autonomous nonlinear term with arbitrary order polynomial components. We term (1) the original equation, which is an ordinary differential equation (ODE), or a partial differential equation (PDE) that has been discretised in space to form a system of ODEs. The parameter $\epsilon \in \mathbb {R}$ measures how rapid the linear oscillations are. The need to sufficiently resolve the fastest of these frequencies on an $\mathcal {O}(\epsilon ^{-1})$ timescale constrains the maximum allowable timestep of explicit timestepping methods in the original equation. This restriction to small timesteps is also true for implicit methods if accuracy is required.

The operator splitting and phase-averaged methods avoid the small timestep restriction in (1) by mapping to a less oscillatory modulation variable, $\textbf{V}$, with use of the matrix exponential. For operator splitting, this mapping is defined as

2 $$\begin{aligned} \textbf{U}(t) = e^{-\frac{t}{\epsilon } \mathcal {L} } \textbf{V}(t). \end{aligned}$$

Using the modulation variable in the original Eq. (1) leads to a new system to timestep,

3 $$\begin{aligned} \frac{\partial \textbf{V}}{\partial t} = e^{\frac{t}{\epsilon } \mathcal {L}} \mathcal {N} \left( e^{- \frac{t}{\epsilon } \mathcal {L}} \textbf{V} \right) , \ \ \ \textbf{V}(0) = \textbf{U}_0. \end{aligned}$$

The original equation can be recovered by applying $\exp (-t\mathcal {L}/\epsilon )$ to (3). Advantageously, the modulation variable system (3) has no linear terms oscillating on an $\mathcal {O}(\epsilon ^{-1})$ timescale, so there is a reduction in numerical stiffness compared to (1). Mapping to a modulation variable equation is standard practice in the method of averaging [35, 36] and has been used to solve stiff PDEs in different fields, including optics [37] and geophysical fluid flows [14–17], and in particular, [8]. The matrix exponential mapping (2) is also a critical component of exponential integrator methods, leading to a variation-of-constants equation for $\textbf{U}$ that is solved numerically by approximating an integral of the nonlinearity [38–40].

A limitation of timestepping the modulation variable Eq. (3) is that there remain oscillations that restrict large timestep stability and accuracy, which is seen when computing higher-order time derivatives. These oscillations can be smoothed by averaging the time evolution of the modulation variable. For example, one can apply classical averaging techniques to remove all oscillations. In the phase-averaging method, a new modulation variable mapping is defined to facilitate a local time average. Following [25] and [41], we introduce a phase variable *s* to specify a time-translation,

4 $$\begin{aligned} \textbf{V}(t,s) = e^{\frac{t + s}{\epsilon } \mathcal {L} } \textbf{U}(t), \end{aligned}$$

so that the phase variable appears in the modulation variable timestepping Eq. (3),

5 $$\begin{aligned} \frac{\partial \textbf{V} (t,s) }{\partial t} = e^{\frac{t + s}{\epsilon } \mathcal {L} } \mathcal {N} \left( e^{- \frac{t + s}{\epsilon } \mathcal {L} } \textbf{V} (t) \right) . \end{aligned}$$

Integrating with respect to the phase variable obtains an averaged system of

6 $$\begin{aligned} \frac{\partial \overline{\textbf{V}}(t)}{\partial t} = \int _{s = - \eta / 2}^{\eta / 2} \mathcal {K}(s)e^{\frac{t + s}{\epsilon } \mathcal {L} } \mathcal {N} \left( e^{- \frac{t + s}{\epsilon } \mathcal {L} } \overline{\textbf{V}} (t) \right) \text {d}s, ~\overline{\textbf{V}}(0) = \textbf{U}_0, \end{aligned}$$

with $\mathcal {K}(s)$ the averaging kernel function and η the averaging window length. Properties of the averaging kernel will be discussed in Sect. 3.1. An important feature of the phase-averaged timestepping Eq. (6) is that the computation occurs at a *fixed* value of the modulation variable, $\overline{\textbf{V}}(t)$, so the averaging only acts on the oscillations induced by the matrix exponential. Back transforming the phase-averaged modulation variable gives the solution in the original variables,

7 $$\begin{aligned} \textbf{U}_{\text {PA}}(t) = e^{-\frac{t}{\epsilon } \mathcal {L}} \overline{\textbf{V}}(t). \end{aligned}$$

The choice of averaging window length η has a significant impact on the phase-averaged solution. A window size of η=0 implies no averaging, so the phase-averaged system (6) returns to the standard timestepping equation for the modulation variable (3), which remains constrained by higher-order oscillations. On the other hand, taking $\eta \rightarrow \infty$ applies a classical time-average, which simulates the asymptotic limit of $\epsilon \rightarrow 0$. A classically averaged solution has a complete cancellation of oscillations; any parts of the solution with periodicity, and thus phase dependence, are driven to zero. As such, only non-oscillatory components contribute to the leading-order solution [19]. [12] showed that the dynamics in the $\epsilon \rightarrow 0$ limit can be replicated numerically with a compactly supported kernel of sufficiently large η.

Phase-averaging, which we focus on, lies between the extremes of no averaging and classical averaging. Its use of a finite η filters the highest frequencies that constrain the explicit timestep size in the unaveraged modulation variable, whilst permitting important low frequency oscillations that are lost with classical averaging. [20] showed that for phase-averaging at a fixed timestep of Δt and in a finite ϵ regime, there is an averaging window of $\eta ^*$ that leads to a solution with minimum error. Insufficient averaging $(\eta < \eta ^*)$ means that the timestepping equations remain too stiff and leaves a significant timestepping error. Excessive averaging $(\eta > \eta ^*)$ removes key dynamical information contained in the oscillations, so there is a dominant averaging error. The combination of timestepping error (decreasing with η) and averaging error (increasing with η) is smallest at the best averaging window of $\eta ^*$. Visualisations of phase-averaged solution errors against η allow the identification of $\eta ^*$; we term these *Peddle plots* [41] (*q.v.* Figs. 3, 6).

The best averaging window depends on the timestep size, as shown in the error bound for phase-averaging [20]. [41] also observed that the simple heuristic of $\eta ^* \approx \Delta t$ can be effective for ϵ=1. Due to the timestep dependence in $\eta ^*$, our Peddle plots and numerical tests will consider a normalised averaging window of

8 $$\begin{aligned} \zeta = \frac{\eta }{\Delta t}. \end{aligned}$$

### Mean corrected phase-averaging

We now introduce the mean corrected phase-averaging method, with the mean correction term having the potential to reduce averaging error in the standard phase-averaging algorithm of [20]. Our mean corrected method follows the approach of [28], with the modification that finite time averages are used to compute a local mean correction, instead of a classical time average that removes all oscillations in the mean correction.

The modulation variable mappings for operator split (2) and phase-averaged (4) methods effectively assume that the linear oscillations, captured in the matrix exponential, are about a state of zero. The idea of [28] is that the transform can be improved by allowing for oscillations around a non-zero mean state. To derive this additional term, we assume that the nonlinearity is slowly varying and can be approximated as constant over one timestep, $\mathcal {N}(\textbf{W}) \sim \textbf{C} (\textbf{W} (t))$, where $\textbf{W}$ is a new modulation variable and $\textbf{C}$ is a local mean of the nonlinearity. We then seek a mean component of the modulation variable, $\textbf{W}_M$, by considering the original Eq. (1) with the constant nonlinearity:

9 $$\begin{aligned} \frac{\partial \textbf{W}_M}{\partial t} + \frac{1}{\epsilon } \mathcal {L} \textbf{W}_M&= \textbf{C}, \nonumber \\ \rightarrow \textbf{W}_M&= \epsilon \mathcal {L}^+ \textbf{C}. \end{aligned}$$

As in [28] we have introduced the Moore-Penrose pseudoinverse of $\mathcal {L}^+$ [42] to deal with the case that $\mathcal {L}$ is singular and $\mathcal {L}^{-1}$ is undefined. We compute the Moore-Penrose inverse using an eigendecomposition of the linear operator as $\mathcal {L}=\mathcal {S}^{-1} \Lambda \mathcal {S}$, with Λ a diagonal matrix containing the eigenvalues $\omega _i$, and $\mathcal {S}$ a matrix containing the eigenvectors. Then, the Moore-Penrose pseudoinverse is computed as $\mathcal {L}^+=\mathcal {S} \Lambda ^+ \mathcal {S}^{-1}$, with the diagonal entries in $\Lambda ^+$ defined as

10 $$\begin{aligned} \Lambda _{ii}^+= {\left\{ \begin{array}{ll} \omega _i^{-1}, & \text {if} ~\omega _i \ne 0, \\ 0, &  \text {if} ~\omega _i = 0. \end{array}\right. } \end{aligned}$$

Adding the mean $\textbf{W}_M$ term (9) to the standard modulation variable mapping (2) obtains the mean corrected mapping,

11 $$\begin{aligned} \textbf{U}(t) = e^{-\frac{t}{\epsilon } \mathcal {L}} \textbf{W}(t) + \epsilon \mathcal {L}^+ \textbf{C}. \end{aligned}$$

The additional $\epsilon \mathcal {L}^+ \textbf{C}$ term can be viewed as a particular integral that is added to a complementary function solution of $\exp (-t\mathcal {L}/\epsilon ) \textbf{W}(t)$ that is used for the standard modulation variable mapping (2).

The application for weakly nonlinear systems in [28] used a classical time-average to compute the mean correction. We call this the classical mean correction, $\textbf{C}_{\infty }$, defined as

12 $$\begin{aligned} \textbf{C}_{\infty } = \lim _{\eta _C \rightarrow \infty } \frac{1}{\eta _C} \int _{r = -\eta _C/2}^{\eta _C/2} \mathcal {N} \left( e^{-\frac{t + r}{\epsilon } \mathcal {L}} \textbf{W}(t) \right) \text {d} r. \end{aligned}$$

In some situations, it is feasible to compute a closed-form expression for $\textbf{C}_{\infty }$ using (12); we call this the classical mean correction. The classical mean correction will be used for the preliminary tests with the swinging spring ODE (Sect. 4) to develop intuition around potential accuracy improvements. However, our primary focus is on using a finite averaging window to compute $\textbf{C}$. The corresponding *local* mean correction has two key advantages over the classical mean correction for inclusion in the phase-averaging method of [20] at finite ϵ:

- It retains low-frequency components of nonlinear oscillation that are lost when $\eta _C \rightarrow \infty$.
- It can be computed algorithmically for any numerical model, instead of being derived for a specific equation and spatial discretisation choice.

The local mean correction is computed with phase-averaging as

13 $$\begin{aligned} \textbf{C} = \int _{r = - \eta _C / 2}^{\eta _C / 2} \mathcal {K}_C(r) \mathcal {N} \left( e^{-\frac{t + r}{\epsilon } \mathcal {L}} \textbf{W}(t) \right) \text {d}r, \end{aligned}$$

with $\mathcal {K}_C (r)$ the mean correction averaging kernel of finite length $\eta _C$, and *r* is a phase variable. We choose $\eta _C$ such that $\partial \textbf{C} / \partial t$ is small.

To apply phase-averaging to the timestepping equation for the mean corrected modulation variable, we introduce the phase variable *s*, analogously to standard phase-averaging in (4), to obtain

14 $$\begin{aligned} \textbf{W}(t, s) = e^{\frac{t+s}{\epsilon } \mathcal {L}} \left[ \textbf{U}(t) - \epsilon \mathcal {L}^+ \textbf{C}(\textbf{W}(t)) \right] . \end{aligned}$$

Using this mapping in the original Eq. (1):

$$\begin{aligned} \frac{\partial }{\partial t} \left( e^{-\frac{t + s}{\epsilon } \mathcal {L}} \textbf{W} + \epsilon \mathcal {L}^+ \textbf{C} \right) + \frac{1}{\epsilon } \mathcal {L} \left( e^{-\frac{t + s}{\epsilon } \mathcal {L}} \textbf{W} + \epsilon \mathcal {L}^+ \textbf{C} \right) = \mathcal {N}, \nonumber \\ \rightarrow -\frac{1}{\epsilon } \mathcal {L} e^{-\frac{t + s}{\epsilon } \mathcal {L}} \textbf{W} + e^{-\frac{t + s}{\epsilon } \mathcal {L}} \frac{\partial \textbf{W}}{\partial t} + \epsilon \frac{\partial }{\partial t} (\mathcal {L}^+ \textbf{C}) + \frac{1}{\epsilon } \mathcal {L} e^{-\frac{t + s}{\epsilon } \mathcal {L}} \textbf{W} + \mathcal {L} \mathcal {L}^+ \textbf{C} = \mathcal {N}, \nonumber \\ \rightarrow e^{-\frac{t + s}{\epsilon } \mathcal {L}} \frac{\partial \textbf{W}}{\partial t} + \epsilon \delta = \mathcal {N} - \mathcal {L} \mathcal {L}^+ \textbf{C}, \nonumber \end{aligned}$$

where

15 $$\begin{aligned} \delta = \frac{\partial }{\partial t} (\mathcal {L}^+ \textbf{C}). \end{aligned}$$

To proceed, we choose an $\eta _C$ such that $\delta \ll 1$, allowing us to neglect the $\epsilon \delta$ term and arrive at

16 $$\begin{aligned} \frac{\partial \textbf{W}(t,s)}{\partial t} = e^{\frac{t + s}{\epsilon } \mathcal {L}} [\mathcal {N} - \mathcal {L} \mathcal {L}^+ \textbf{C}]. \end{aligned}$$

This equation is phase-averaged over a finite time window to give the following numerical system for $\overline{\textbf{W}}$,

17 $$\begin{aligned} \begin{aligned} \frac{\partial \overline{\textbf{W}} (t)}{\partial t} = \int _{s = - \eta / 2}^{\eta / 2} \mathcal {K}(s) e^{ \frac{t+s}{\epsilon } \mathcal {L}} \left[ \mathcal {N} \left( e^{ - \frac{t+s}{\epsilon } \mathcal {L}} \overline{\textbf{W}} + \epsilon \mathcal {L}^+ \textbf{C} \right) - \mathcal {L} \mathcal {L}^+ \textbf{C} \right] \text {d} s, \\ \overline{\textbf{W}}(0) = \textbf{U}_0 - \epsilon \mathcal {L}^+ \textbf{C} (\overline{\textbf{W}}(0)). \end{aligned} \end{aligned}$$

Back transforming the mean corrected phase-averaged modulation variable gives the solution in the original variables,

18 $$\begin{aligned} \textbf{U}_{\text {MC}}(t) = e^{-\frac{t}{\epsilon } \mathcal {L}} \mathbf {\overline{W}}(t) + \epsilon \mathcal {L}^+ \textbf{C}(\overline{\textbf{W}}(t)). \end{aligned}$$

We note two features of the mean corrected phase-averaged system (17). First, the initial condition $\overline{\textbf{W}}(0)$ is defined implicitly, and our approach for computing this will be discussed in Sect. 3, along with other algorithmic details. Second, in systems where the linear operator is invertible, $\mathcal {L}\mathcal {L}^+ = \mathcal {L}\mathcal {L}^{-1}=I$, where *I* is the identity; this simplifies the $\mathcal {N} - \mathcal {L} \mathcal {L}^+ \textbf{C}$ term to $\mathcal {N} - \textbf{C}$. However, when $\mathcal {L}$ is singular, it is generally the case that $\mathcal {L}\mathcal {L}^+ \ne I$.

### A simple example with the mean correction

We now provide intuition around the mean corrected version of phase-averaging through an ODE example. Using the idea of [28], we consider the simplest ‘nonlinearity’ of $\mathcal {N} = c$, for a constant *c*. Our example differs from that of [28] in that we consider finite phase-averaging in an oscillatory multiscale system. In this simple ODE, we can compute an analytical solution to compare the accuracy of mean corrected and standard phase-averaging (Fig. 1).

Consider an original form (1) nondimensional ODE for a single variable, with an oscillation frequency ω and constant *c*,

19 $$\begin{aligned} \frac{\text {d} {U}}{\text {d}t} + \frac{1}{\epsilon } i \omega U = c, ~U(0)=U_0. \end{aligned}$$

The modulation variable transform ($\textbf{U} = \exp (-t \mathcal {L}/\epsilon ) \textbf{V}$) is

20 $$\begin{aligned} U = e^{- \frac{i \omega t}{\epsilon }} V, \end{aligned}$$

which leads to an initial value problem of

21 $$\begin{aligned} \frac{\text {d} {V}}{\text {d}t} = e^{\frac{i \omega t}{\epsilon }} c, ~V(0)=U_0. \end{aligned}$$

This can be solved in the modulation variable space and back-transformed to obtain the analytical solution of

22 $$\begin{aligned} U(t) = \frac{i \epsilon c}{\omega } \left( e^{-\frac{i \omega t}{\epsilon }} - 1 \right) + e^{-\frac{i \omega t}{\epsilon }} U_0. \end{aligned}$$

Now, consider the application of standard phase-averaging to the modulation variable system (21),

23 $$\begin{aligned} \frac{\text {d} {\overline{V}}}{\text {d}t} = \chi e^{\frac{i \omega t}{\epsilon }} c, ~\overline{V}(0)=U_0, \end{aligned}$$

with χ denoting a finite phase-average of the matrix exponential,

24 $$\begin{aligned} \chi = \int _{s = - \eta / 2}^{\eta / 2} \mathcal {K}(s) e^{\frac{i \omega s}{\epsilon }} \text {d}s. \end{aligned}$$

When η=0, $\mathcal {K}(s)$ collapses to a dirac delta function, leaving χ=1 and the phase-averaged timestepping Eq. (23) returns to that of the unaveraged modulation variable (21). Applying some level of phase-averaging, η>0, leads to χ<1 and a reduction in numerical stiffness that is beneficial for taking larger explicit timesteps. Solving the phase-averaged system (23) leads to a modulation variable solution of

25 $$\begin{aligned} \overline{V}(t) = \chi \frac{i \epsilon c}{\omega } \left( 1 - e^{\frac{i \omega t}{\epsilon }} \right) + U_0, \end{aligned}$$

and an original space phase-averaged solution of

26 $$\begin{aligned} U_{\text {PA}}(t) = \chi \frac{i \epsilon c}{\omega } \left( e^{-\frac{i \omega t}{\epsilon }} - 1 \right) + e^{-\frac{i \omega t}{\epsilon }} U_0. \end{aligned}$$

The averaging error with standard phase-averaging is therefore

27 $$\begin{aligned} U(t) - U_{\text {PA}}(t) = (1 - \chi ) \frac{i \epsilon c}{\omega } \left( e^{-\frac{i \omega t}{\epsilon }} - 1 \right) . \end{aligned}$$

This result exemplifies the characteristic trade-off of the phase-averaging method: a larger averaging window has a greater reduction in numerical stiffness (24) and the associated timestepping error, but at the cost of increased averaging error.

We now turn to the mean corrected method to reduce the averaging error. In this example, the local mean correction equals the constant *c* for any choice of mean correction averaging window, $\eta _C$. The mean corrected mapping ($\textbf{U} = \exp (-t\mathcal {L}/\epsilon ) \textbf{W} + \epsilon \mathcal {L}^+ \textbf{C}$) is defined as

28 $$\begin{aligned} U = e^{- \frac{i \omega t}{\epsilon }} W - \epsilon \frac{i}{\omega } c, \end{aligned}$$

with initial condition

29 $$\begin{aligned} W_0 = U_0 + \frac{i \epsilon c}{\omega }. \end{aligned}$$

The corresponding mean corrected system (17) is,

30 $$\begin{aligned} \frac{\text {d} {\overline{W}}}{\text {d}t} = \int _{s = - \eta / 2}^{\eta / 2} \mathcal {K}(s) e^{\frac{i \omega (t+s)}{\epsilon }} [c - c] ~\text {d} s = 0. \end{aligned}$$

Hence, the new modulation variable is constant in time, $\overline{W}(t) = W_0$. Note, this has not required the approximation of $\delta \ll 1$ (15).

With a back-transformation, the mean corrected solution in the original space is

31 $$\begin{aligned} U_{\text {MC}}(t) = \frac{i \epsilon c}{\omega } \left( e^{-\frac{i \omega t}{\epsilon }} - 1 \right) + e^{-\frac{i \omega t}{\epsilon }} U_0. \end{aligned}$$

This is precisely the analytical solution of (22), meaning that the mean corrected method is free of averaging error in this example.

To visualise the difference between the phase-averaging methods in this simple ODE, Fig. 1 shows an instance where $\epsilon =0.143, ~\omega =2\pi , ~c=5, u_0=1+i$, and the simulation end time is $T_{\text {end}}=1$. Numerical phase-averaged solutions, with the standard and mean corrected methods, are computed with explicit RK4 and a timestep of Δt=0.2. The averaging window of $\zeta =1 \rightarrow \eta =0.2$ leads to a stiffness reduction factor (24) of χ=0.101 for standard phase-averaging. This reduction in numerical stiffness allows larger and more accurate timesteps for explicit methods. However, it also introduces averaging error in both the real and imaginary components of *U*(*t*), which is most clearly visualised in the absolute value of the solution, $|U_{\text {PA}}(t)|$ (Fig. 1b). By contrast, the mean corrected method exactly obtains the analytical solution at each timestep, due to a modified mapping such that *W* is constant in time and is unaffected by averaging error.

This example shows that the information contained within the mean correction can allow for smaller averaging errors with explicit timestepping. We will soon test whether this improved accuracy holds in more complex differential equations that require numerical simulation.

## Algorithmic implementation

We now discuss some important algorithmic components of phase-averaging and the mean corrected method.

### Averaging kernels

Phase-averages are computed numerically using the averaging kernel $\mathcal {K}(s)$. Necessary properties of the averaging kernel are discussed in [41], with an exponential bump function selected to satisfy these. This choice ensures sufficient smoothness when approximating the $\epsilon \rightarrow 0$ limit with a compact support [12]. The kernel has a maximum value at no phase shift (s=0) and decays to zero at the boundaries of the averaging interval,

32 $$\begin{aligned} \mathcal {K}(s) = {\left\{ \begin{array}{ll} A \exp \left( \frac{-\eta ^2}{\Gamma (0.5 \eta + s) (0.5 \eta - s)} \right) , & s \in \left( -\frac{\eta }{2},\frac{\eta }{2} \right) , \\ 0, & |s| \ge \frac{\eta }{2}, \end{array}\right. } \end{aligned}$$

where *A* is a normalisation constant such that $\int _{s=-\eta /2}^{\eta /2} \mathcal {K}(s) \ \text {d} s = 1$. A kernel decay rate of Γ controls the shape of the kernel over the compact support. Values of Γ=1 [20, 24] and Γ=4 [41] have been used in prior applications — we use the latter for the results in this paper.

For numerical implementation, the phase-averaged timestepping Eq. (6) is expressed as a sum of evaluations at $K_s$ equispaced phase shifts,

33a $$\begin{aligned} \frac{\partial \overline{\textbf{V}} (t)}{\partial t}&= \sum _{p=1}^{K_s} \mathcal {K}(s_p) e^{ \frac{t+s_p}{\epsilon } \mathcal {L}} \mathcal {N} \left( e^{- \frac{t+s_p}{\epsilon } \mathcal {L}} \overline{\textbf{V}}(t) \right) , \end{aligned}$$

33b $$\begin{aligned} s_p&= \left( \frac{2p-1}{2K_s} - \frac{1}{2} \right) \eta , \end{aligned}$$

where the discrete kernel values are normalised such that $\sum _{p=1}^{K_s} \mathcal {K}(s_p) = 1$.

Convergence of the numerical phase-average requires a sufficient number of discrete kernel points, $K_s$. For PDE systems, $K_s$ scales with the fastest resolvable frequency and the averaging window length. We choose

34 $$\begin{aligned} K_s = \Big \lceil P \eta \frac{\omega _{\text {max}}}{2 \pi \epsilon } \Big \rceil , \end{aligned}$$

where $\lceil . \rceil$ denotes the ceiling function, *P* is the number of samples within each period of this fastest frequency, and $\omega _{\text {max}}$ is the fastest resolvable (angular) frequency of $\mathcal {L}$. To have sufficient sampling of the fastest oscillations, a value of at least P=3.5 is recommended for pseudospectral methods [43]. Following [24], we use P=4. As a proportionally larger number of points per oscillation may be required when sampling fewer oscillations [43], a minimum $K_s$ value is also specified. As the numerical phase-average in (33) is computed at a fixed value of the modulation variable, $\overline{\textbf{V}}(t)$, each evaluation at phase shift $s_p$ is independent, which allows the phase-average to be computed in parallel using $K_s$ processors [12].

The kernel for the local mean correction, $\mathcal {K}_C(r)$, uses the same definition as $\mathcal {K}(s)$ (32). The required $K_r$ number of points in $\mathcal {K}_C(r)$ is computed using (34) with $\eta _C$ in place of η. As with the phase-average of a timestepping equation, i.e. (6) or (17), the local mean correction is computed at a fixed solution state, $\overline{\textbf{W}}(t)$, so it is parallelisable. Algorithm 1 provides pseudocode for the computation of the local mean correction.

**Algorithm 1:**
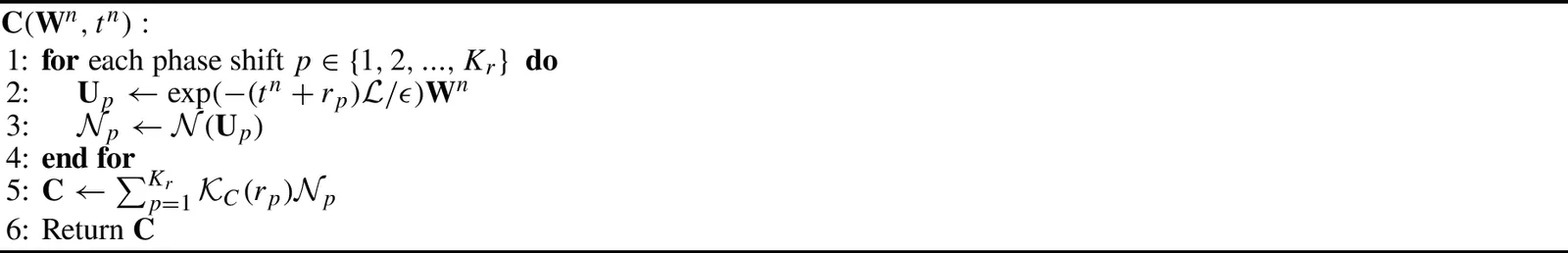
The local mean correction

### The mean corrected phase-averaging algorithm

Algorithm 2 describes the locally mean corrected phase-averaging algorithm that solves (17). Phase-averaging operations, for both the timestepping equations and local mean correction, can be computed in parallel. However, as this paper is concerned with exploring potential accuracy improvements, not parallelisation, our numerical experiments use a serial implementation. The locally mean corrected method requires the selection of a suitable pair of averaging windows, $(\eta , \eta _C)$, and our approach for identifying this in the RSWEs experiments will be discussed in Sect. 5. Analogously to an $\eta ^*$ minimising the error of the phase-averaging method, the existence of a finite $\eta _C^*$ which minimises the error of the locally mean corrected method is observed in our RSWEs experiments.

The initial condition for the mean corrected modulation variable (17) is defined implicitly, and we compute this with the fixed-point iteration of Algorithm 3, starting from a guess of $\textbf{W}_0 = \textbf{U}_0$. This implicit equation only needs to be solved at the initial time, as afterwards the solution is timestepped in the $\textbf{W}$ space and back transformations to $\textbf{U}$ (11) are defined explicitly.

**Algorithm 2:**
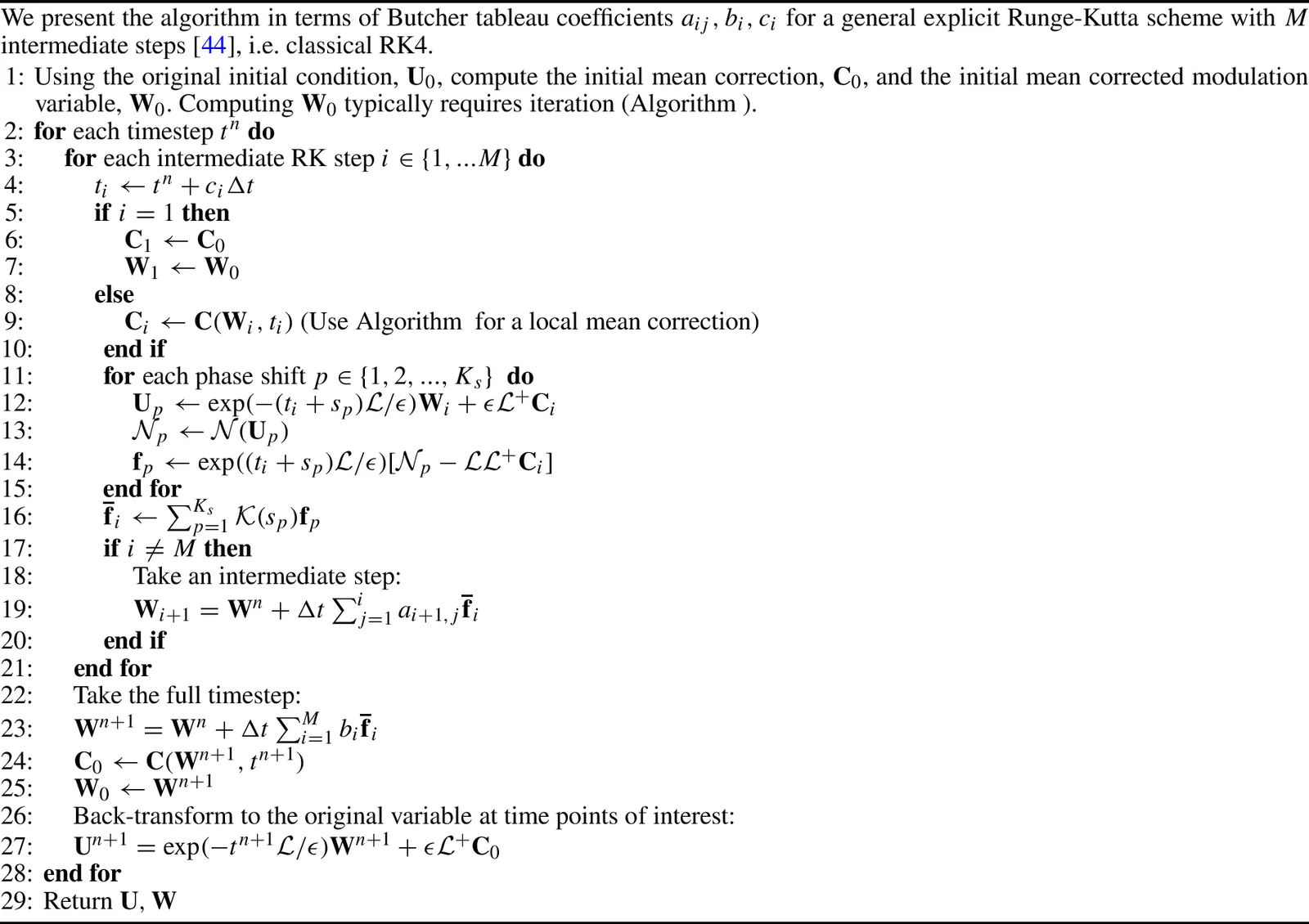
The mean-corrected method

**Algorithm 3:**
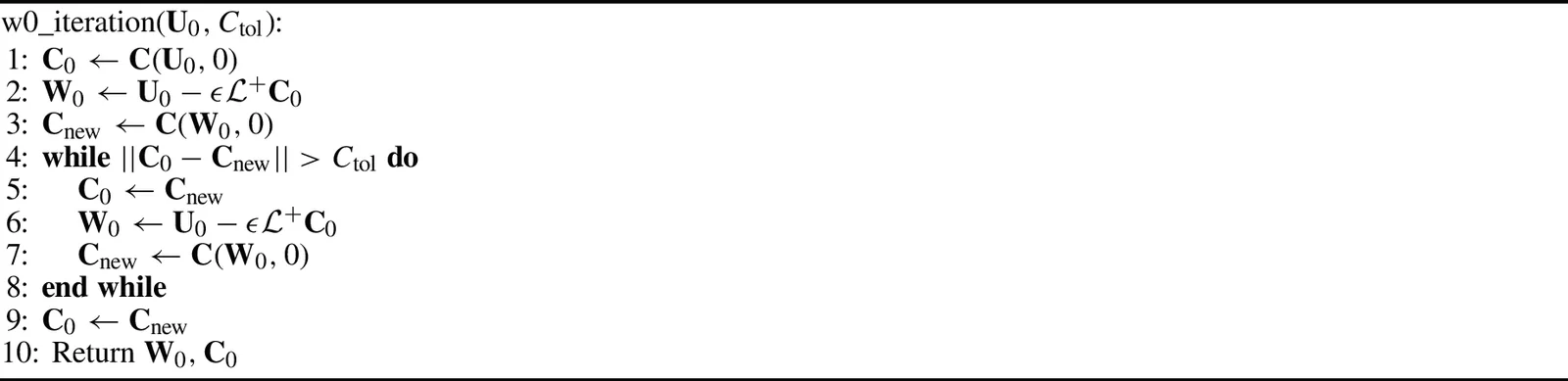
Fixed-point iteration for computing $\textbf{W}(0)$

In this proof-of-concept study of the mean corrected phase-averaging method, our focus is on potential accuracy improvements. For practical implementation and computational cost, we sketch three topics whose details are beyond the scope of this paper. First, the mean corrected method requires more computation than standard phase-averaging, due to the additional averaging operation to compute the local mean correction (13). However, as phase-averaging methods often enable larger timesteps, it is complicated to evaluate relative computational costs. For example, the number of points used in the averaging kernels, the averaging window lengths, the increase in timestep size with phase-averaging, and the approach for storing the phase-average evaluations will all factor into this cost calculation. Second, because phase-averaging increases computation in the time dimension only, the method generalises to higher spatial dimensions, i.e. 2D and 3D. Whilst this work uses smaller problems to investigate accuracy improvements, detailed estimates of the computational cost and memory bandwidth require future investigation. Third, whilst the initial condition iteration of Algorithm 3 is inexpensive in our numerical tests, the generalisability to arbitrary initial conditions requires further analysis.

## The swinging spring ODE

The first numerical experiment we consider is the swinging spring ODE. This system has been shown to be a simplified model of nonlinear resonances involving fast inertia-gravity waves and slow Rossby waves in the RSWEs [45]. The swinging spring ODE contains two physical modes which exhibit fast, oscillatory dynamics: swinging with a frequency of $\omega _R$, and elastic springing with a frequency of $\omega _Z$. To identify the dynamics of the system, we introduce a *resonance factor* parameter as the ratio of the normal mode frequencies,

35 $$\begin{aligned} \rho =\frac{\omega _Z}{\omega _R}. \end{aligned}$$

Varying ρ mimics different types of wave interaction that occur in the RSWEs (Sect. 5). Furthermore, one can exactly describe the time evolution of hyperbolic PDEs with a bilinear $\mathcal {N}$, such as the RSWEs, as the sum of all resolvable interactions of three linear waves [41, 46]. As a corollary, interactions of fast linear waves can construct slow nonlinear dynamics [22, 23, 47].

We consider three regimes of normal mode interaction in the swinging spring:

- Direct resonance, where $\rho \in \mathbb {Z}$.
- Near resonance, where ρ is close to being an integer.
- Far resonance, where ρ is not close to being an integer.

Previous swinging spring studies have focused on the directly resonant case of ρ=2, e.g. [29, 48, 49], and with higher-order phase-averaging in [25]. The direct resonance leads to a slow timescale stepwise precession, where the angle of the pendulum in the *x*-*y* plane changes sharply at fixed intervals of time [29]. One full cycle of the nonlinear phase precession occurs on a much slower timescale than the swinging and springing modes, corresponding to $\epsilon \approx 0.01$ [25]. In preparation for the RSWEs, which is a PDE system containing a spectrum of direct, near, and far resonance interactions, we examine $\rho \in [1.5,2.5]$.

### Equations

We use the governing equations in [29], which were derived using a cubic-order truncated Lagrangian for the pendulum’s position as a function of time,

36a $$\begin{aligned} \frac{\text {d}^2 x}{\text {d}t^2} + \omega _R^2 x&= \lambda xz, \end{aligned}$$

36b $$\begin{aligned} \frac{\text {d}^2 y}{\text {d}t^2} + \omega _R^2 y&= \lambda yz, \end{aligned}$$

36c $$\begin{aligned} \frac{\text {d}^2 z}{\text {d}t^2} + \omega _Z^2 z&= \frac{\lambda }{2}(x^2 + y^2), \end{aligned}$$

where $\lambda = l_0 \omega _Z^2 / l^2$, for equilibrium and unstretched lengths *l* and $l_0$, respectively. We also have the relationship between the two linear frequencies of $\omega _Z = \rho \omega _R$ (35).

Following [25], the following transformation is applied to form a first-order system in time,

37 $$\begin{aligned} \frac{\text {d} {x}}{\text {d}t} = \omega _R p_x, \ \frac{\text {d} {y}}{\text {d}t} = \omega _R p_y, \ \frac{\text {d} {z}}{\text {d}t} = \omega _Z p_z, \end{aligned}$$

introducing $p_a = \partial p /\partial a, ~a \in \{x,y,z\}$. To work with three variables instead of six, we use a complex-valued solution vector of $\textbf{U} = [\overset{\circ }{x},\overset{\circ }{y},\overset{\circ }{z}]^\text {T}$, with

38 $$\begin{aligned} \overset{\circ }{x} = x + i p_x, \ \overset{\circ }{y} = y + i p_y, \ \overset{\circ }{z} = z + i p_z. \end{aligned}$$

Taking the real part of each component of $\textbf{U}$ recovers the position in that coordinate. Using the resonance factor of ρ (35) to relate the swinging and springing modes, we obtain the swinging spring in the original equation form (1), with operators

39 $$\begin{aligned} \mathcal {L} = \begin{bmatrix} i \omega _R &  0 &  0 \\ 0 &  i \omega _R &  0 \\ 0 &  0 &  i \rho \omega _R \\ \end{bmatrix}, \ \mathcal {L}^{-1} = \begin{bmatrix} \frac{-i}{\omega _R} &  0 &  0 \\ 0 &  \frac{-i}{\omega _R} &  0 \\ 0 &  0 &  \frac{-i}{\rho \omega _R} \\ \end{bmatrix}, \ \mathcal {N} = \begin{bmatrix} \frac{i \lambda }{\omega _R} \text {Re} (\overset{\circ }{x}) \text {Re} (\overset{\circ }{z}) \\ \frac{i \lambda }{\omega _R} \text {Re} (\overset{\circ }{y}) \text {Re} (\overset{\circ }{z}) \\ \frac{i \lambda }{2 \rho \omega _R} [\text {Re} (\overset{\circ }{x})^2 + \text {Re} (\overset{\circ }{y})^2 ] \end{bmatrix}. \end{aligned}$$

As $\mathcal {L}$ is diagonal, the matrix exponential is simply the exponentiation of the nonzero elements,

40 $$\begin{aligned} e^{\pm t \mathcal {L}} = \begin{bmatrix} e^{ \pm i \omega _R t} &  0 &  0 \\ 0 &  e^{ \pm i \omega _R t} &  0 \\ 0 &  0 &  e^{\pm i \rho \omega _R t} \\ \end{bmatrix}. \end{aligned}$$

The swinging swing problem is directly related to Rossby wave triads [45], which are already in the small ϵ limit. Hence, ϵ does not appear in these equations. For implementation purposes, we use ϵ=1 in Algorithm 2 for the mean corrected method.

### A classical mean correction

We consider a classical mean correction (12) instead of a local mean correction (13) in this first test. This is because the corresponding analytical expression for $\textbf{C}_{\infty }$ provides a physical interpretation and allows more insight as to potential accuracy improvements with a mean correction. It is also feasible to compute $\textbf{C}_{\infty }$ in this simple ODE, which is not typically the case for PDE systems.

Consider the nonlinear vector in terms of the mean corrected modulation variable, $\textbf{W} = [\tilde{x},\tilde{y},\tilde{z}]^\text {T}$,

41 $$\begin{aligned} \mathcal {N}(e^{-t \mathcal {L} }\textbf{W}) = \begin{bmatrix} \frac{i \lambda }{\omega _R} \text {Re} (e^{-i \omega _R t} \tilde{x}) \text {Re} (e^{-i \rho \omega _R t} \tilde{z}) \\ \frac{i \lambda }{\omega _R} \text {Re} (e^{-i \omega _R t} \tilde{y}) \text {Re} (e^{-i \rho \omega _R t} \tilde{z}) \\ \frac{i \lambda }{2 \rho \omega _R} [\text {Re} (e^{-i \omega _R t} \tilde{x})^2 + \text {Re} (e^{-i \omega _R t} \tilde{y})^2 ] \end{bmatrix}. \end{aligned}$$

Using that $\text {Re} ( AB ) = (1/2) (AB + A^* B^* )$, where $^*$ denotes complex conjugation, arrives at

42 $$\begin{aligned} \mathcal {N}(e^{-t \mathcal {L} }\textbf{W}) = \begin{bmatrix} \frac{i \lambda }{4 \omega _R} (A \tilde{x} \tilde{z} + B \tilde{x} \tilde{z}^* + C \tilde{x}^* \tilde{z} + D \tilde{x}^* \tilde{z}^*) \\ \frac{i \lambda }{4 \omega _R} (A \tilde{y} \tilde{z} + B \tilde{y} \tilde{z}^* + C \tilde{y}^* \tilde{z} + D \tilde{y}^* \tilde{z}^*) \\ \frac{i \lambda }{8 \rho \omega _R} (E (\tilde{x}^2 + \tilde{y}^2) + 2(\tilde{x}\tilde{x}^* + \tilde{y}\tilde{y}^*) + F (\tilde{x}^{*2} + \tilde{y}^{*2}) ) \end{bmatrix}, \end{aligned}$$

with

43 $$\begin{aligned} \begin{aligned} A = e^{-i (\rho + 1) \omega _R t}, \ B = e^{i (\rho - 1) \omega _R t}, \ C = e^{-i (\rho - 1) \omega _R t}, \\ D = e^{i (\rho + 1) \omega _R t}, \ E = e^{-2 i \omega _R t}, \ F = e^{2i \omega _R t}. \end{aligned} \end{aligned}$$

For the range of resonance factors considered, all the complex exponential terms (43) have a nonzero argument. A cancellation of oscillations occur in the classical limit of $\eta _C \rightarrow \infty$ (12) so that each of these terms has a zero mean value (refer to the appendix for an example of this). As a result, the only nonzero term is in the *z* coordinate,

44 $$\begin{aligned} \textbf{C}_{\infty } = \begin{bmatrix} 0\\ 0 \\ \frac{i \lambda }{4 \rho \omega _R} (\tilde{x}\tilde{x}^* + \tilde{y}\tilde{y}^*) \end{bmatrix}. \end{aligned}$$

This classical mean correction has a physical interpretation: the positive definite quantity of $\tilde{x} \tilde{x}^* + \tilde{y} \tilde{y}^* = |\tilde{x}|^2 + |\tilde{y}|^2$ is the squared radial position of the pendulum, *within* the modulation variable space. We note that there may be a link between $\textbf{C}_{\infty }$ and the conserved quantities identified for the swinging spring at 2:1 resonance (ρ=2) in [29].

As the only non-zero mean correction component is in the *z* coordinate, the initial states of $\textbf{U}(0)$ and $\textbf{W}(0)$ have the same *x* and *y* components. This allows the mean corrected initial condition to be defined explicitly from the original initial condition, $\textbf{U}_0 = [\hat{x}_0,\hat{y}_0,\hat{z}_0]^\text {T}$, as

45 $$\begin{aligned} \textbf{W}(0) = \textbf{U}_0 - \mathcal {L}^{-1} \textbf{C}_{\infty }(\textbf{U}_0) = \begin{bmatrix} \overset{\circ }{x}_0 \\ \overset{\circ }{y}_0 \\ \overset{\circ }{z}_0 - \frac{\lambda }{4 \rho ^2 \omega _R^2} (\overset{\circ }{x}_0 \overset{\circ }{x}_0 ^* + \overset{\circ }{y}_0 \overset{\circ }{y}_0 ^*) \\ \end{bmatrix}. \end{aligned}$$

### Numerical implementation

Following [45], we set $\omega _R = \pi ~\text {s}^{-1}$ and $\lambda = 1.2 \omega _Z^2 ~\text {s}^{-2}$, with $\omega _Z = \rho \omega _R$ (35). The initial condition is $\textbf{U}(0) = [0.04, 0.03427 i / \pi ,0.08]$ and the simulations are run for a total time of 200 s. The classical explicit fourth-order Runge–Kutta scheme (RK4) is used, with a large timestep of $\Delta t = 0.5 ~\text {s}$ and the operators in (39). Standard phase-averaging solves (6) and the mean corrected phase-averaging method follows Algorithm 2. Errors are measured as the time-averaged L2 error in coordinate position, relative to a reference solution with RK4 and $\Delta t = 0.01 ~\text {s}$.

### Results

**Fig. 2:**
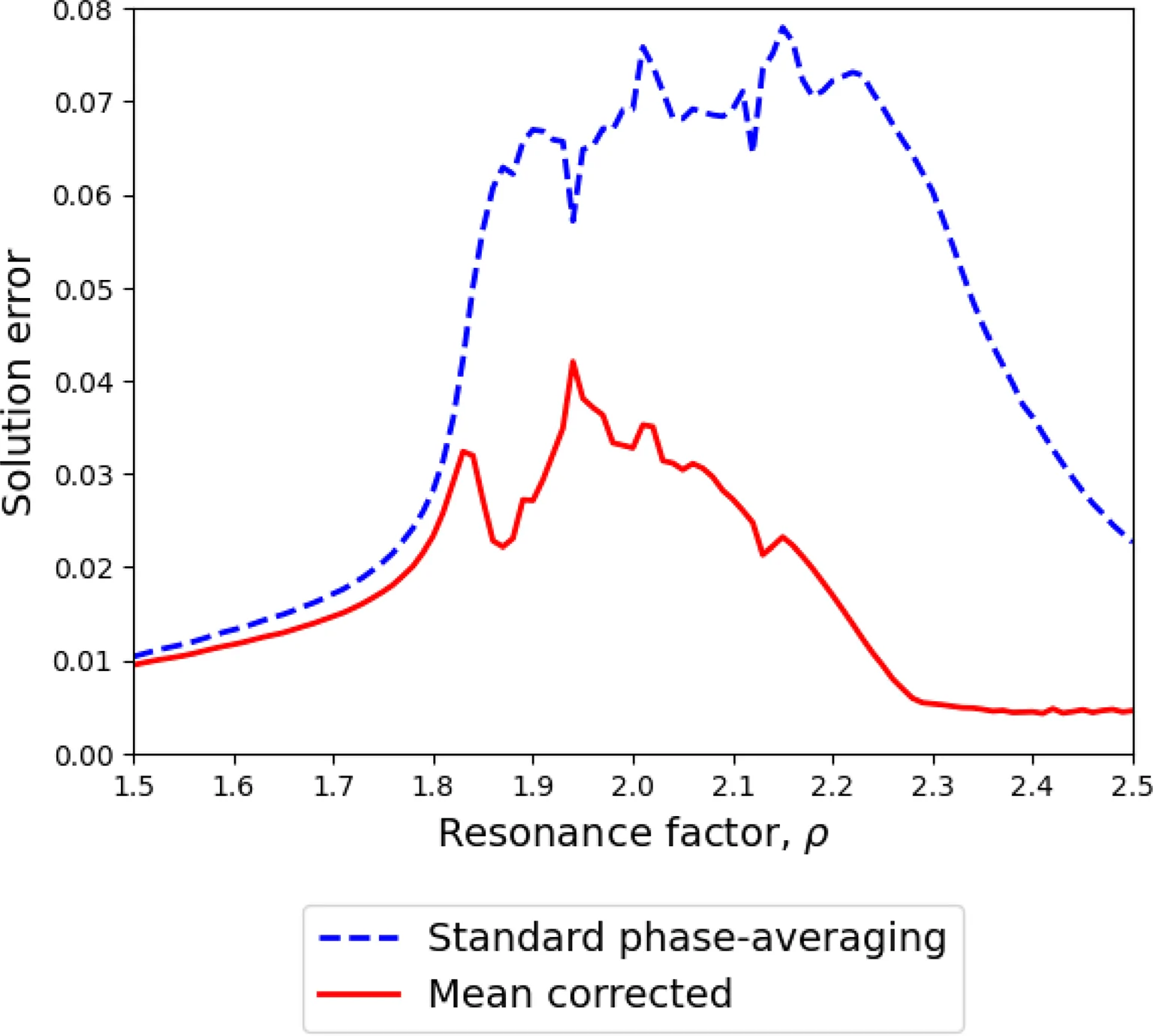
Errors with standard and mean corrected phase-averaging in the swinging spring experiment. The mean corrected method is more accurate for all the resonance factors examined

Errors at the best averaging window for each phase-averaging method are shown in Fig. 2. The mean corrected method has a lower error for all resonance factors, so it improves accuracy in the direct, near, and far resonance configurations of the swinging spring. The accuracy improvement is greater for ρ>1.82, highlighting that the mean correction may have a larger impact for certain regimes of nonlinear dynamics.

**Fig. 3:**
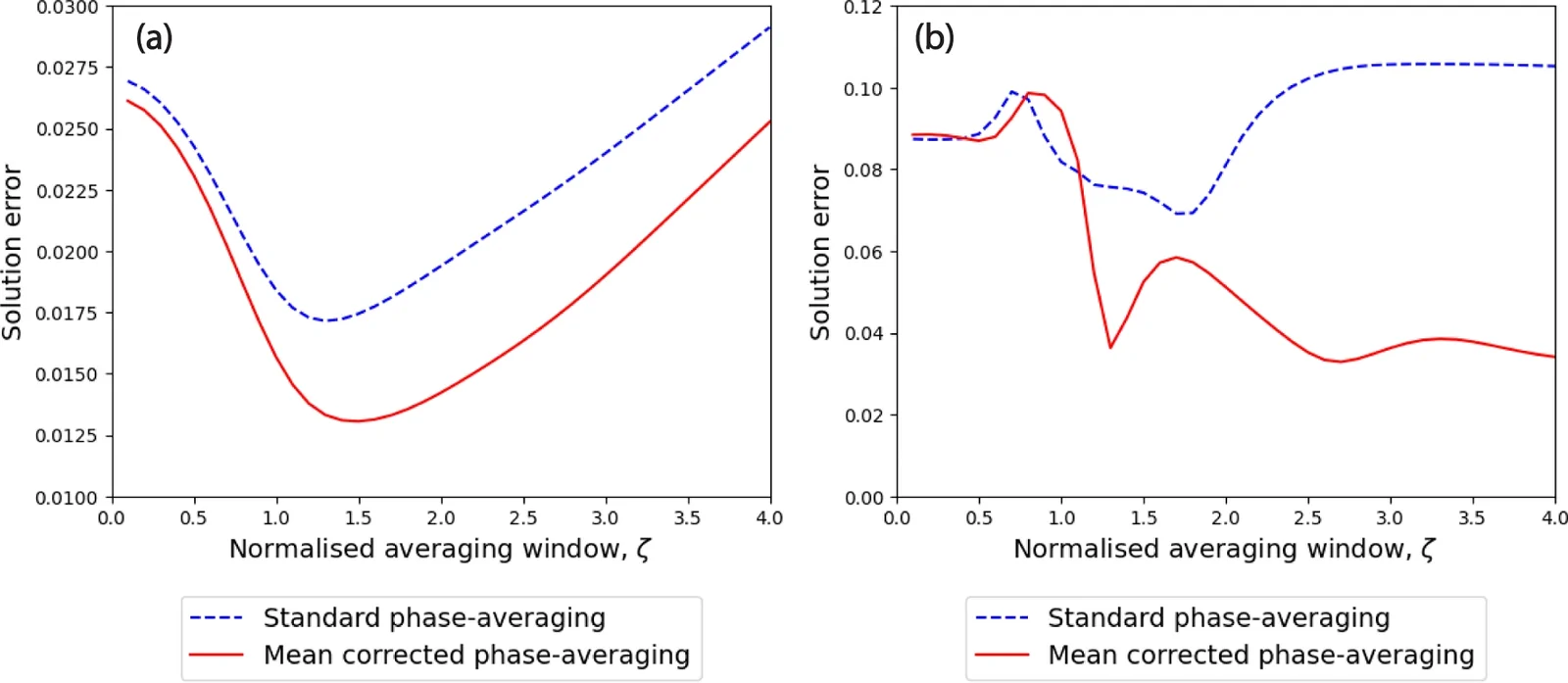
Peddle plots of phase-averaged solution error against the normalised averaging window of $\zeta = \eta /\Delta t$. In an out-of-resonance case (**a**), there is a clear minimum error for each method, with the best window for the mean corrected method being slightly larger. For the directly resonant case (**b**), the mean corrected method again is best with a larger window than standard phase-averaging, and there are also multiple local minima in its error

The best averaging windows are identified from the lowest error of each method over a range of averaging windows separated by $\Delta \zeta = 0.1$, with $\zeta = \eta / \Delta t$. The existence of best averaging windows of $\eta ^*$ is seen in corresponding Peddle plots (Fig. 3). The mean corrected method can have multiple local minima in its Peddle plots, as is the case for ρ=2 (Fig. 3b), whereas standard phase-averaging has a single minimum for each resonance factor.

**Fig. 4:**
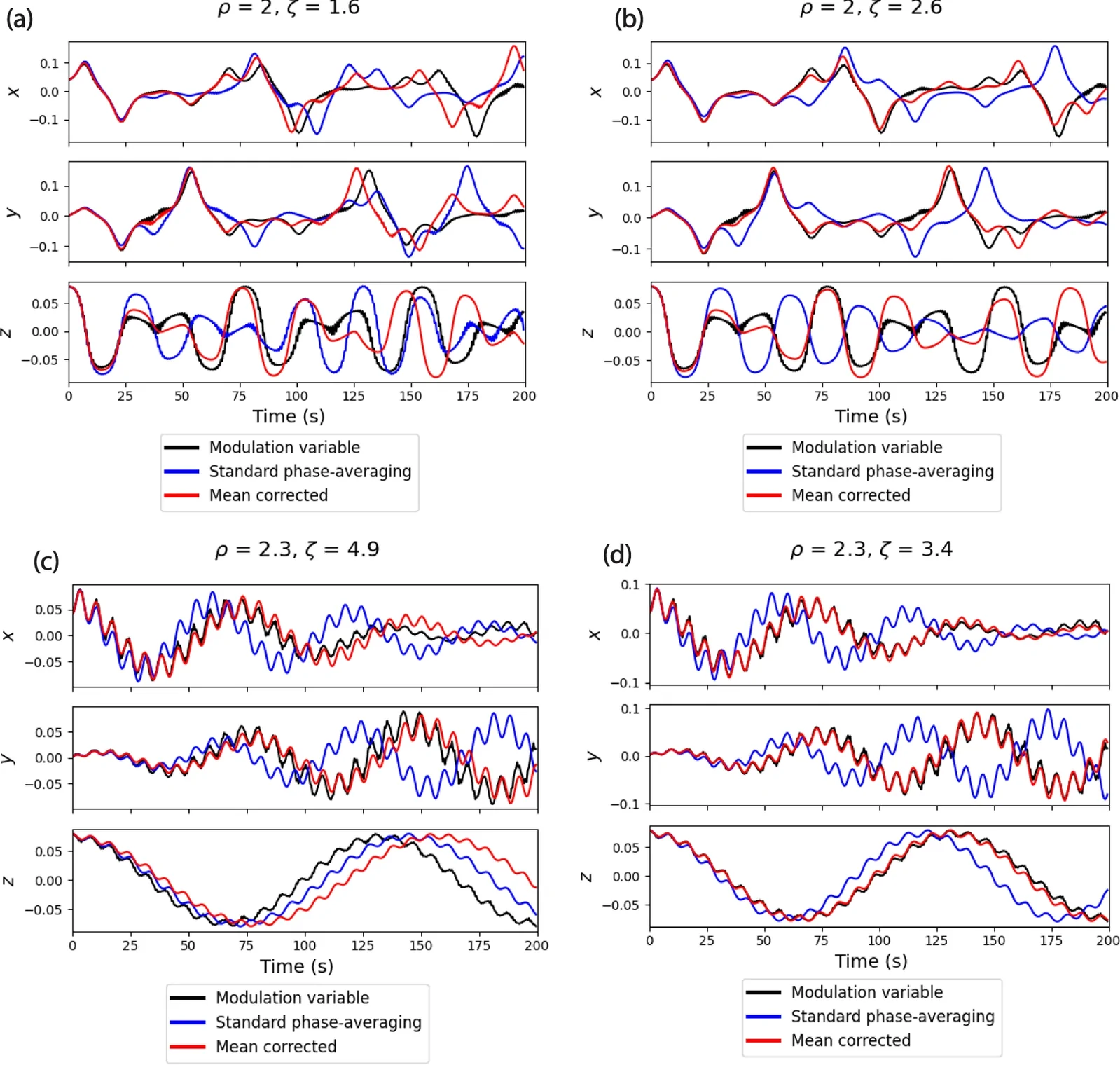
Comparing modulation variable space solutions with standard phase-averaging ($\overline{\textbf{V}}$, blue) and mean corrected phase-averaging ($\overline{\textbf{W}}$, red), with an unaveraged small timestep solution ($\textbf{V}$, black). The directly resonant state of ρ=2 is shown in (**a**) and (**b**), and a far resonant state of ρ=2.3 in (**c**) and (**d**). The modulation variable solutions in (**a**) and (**c**) use the best ζ for standard phase-averaging, whilst (**b**) and (**d**) use the best ζ for the mean corrected method. The mean corrected method aligns better in the modulation space with its best averaging window, whereas standard phase-averaging has considerable phase discrepancies even when using its best window. For the ρ=2.3 case, the maximum error in a single coordinate for the mean corrected method is 0.0139 (**d**), whilst the maximum coordinate error for standard phase-averaging (**c**) is 0.1740

The effect of the mean correction can be observed when comparing modulation variable spaces in Fig. 4. An unaveraged modulation variable solution using (3) represents the ‘true’ modulation variable solution. This contains small amplitude fluctuations, which are reduced with standard phase-averaging. However, the phase-averaging introduces an error, which presents itself as a phase discrepancy in the modulation space, even at the best window for standard phase-averaging, e.g. for ρ=2 in Fig. 4a. With a larger averaging window (Fig. 4b), the standard phase-averaged solution is smoother, but the phase errors are more prevalent, with the *z* coordinate solution sometimes being 180 degrees out of phase. By contrast, the mean corrected method has a much lower phase error using its best $\eta ^*$. Hence, the information provided by the mean correction increases the accuracy within the modulation variable space itself. Figures 4c and 4d again show a phase adjustment with the mean correction, this time for the far resonant case of ρ=2.3.

## Rotating shallow water equations

We now apply the mean corrected method to the rotating shallow water equations (RSWEs). We consider the *f*-plane RSWEs with periodic boundary conditions, like in [8]. The spatial domain is a one-dimensional slice, as was used to test previous phase-averaging algorithms [12, 20, 26]. We use the local version of the mean correction, which is computed numerically (Algorithm 1).

### Equations

The prognostic variables of the RSWEs are a two-dimensional fluid velocity of (*u*, *v*) and a measure of elevation of the fluid layer, such as a height perturbation, $h^{'}$, above the mean fluid height of $H_0$. We consider the RSWEs in the nondimensional form detailed in [41] for a one-dimensional domain with spatial coordinate *x*. To obtain a skew-symmetric linear operator, we use a symmetrical geopotential of $\phi = \sqrt{g / H_0} ~h^{'}$, where *g* is the gravitational acceleration. This leads to an equation set of

46a $$\begin{aligned}&\frac{\partial u}{\partial t} - \frac{1}{\text {Ro}} v + \frac{1}{\text {Fr}} \frac{\partial \phi }{\partial x} + u \frac{\partial u}{\partial x} = 0, \end{aligned}$$

46b $$\begin{aligned}&\frac{\partial v}{\partial t} + \frac{1}{\text {Ro}} u + u \frac{\partial v}{\partial x} = 0, \end{aligned}$$

46c $$\begin{aligned}&\frac{\partial \phi }{\partial t} + \frac{1}{\text {Fr}} \frac{\partial u}{\partial x} + \frac{\partial }{\partial x} (u \phi ) = 0, \end{aligned}$$

with the Rossby number ($\text {Ro}$) quantifying the strength of inertia to the rotational force, and the Froude number ($\text {Fr}$) relating the strength of inertia to the gravitational (restorative) force. We consider a system where the rotational and gravitational forces are balanced by setting $\text {Ro} = \text {Fr} = \epsilon$, which means that the 1D RSWEs (46) are in the original equation form (1).

Representing the RSWEs in Fourier space, where $\hat{\textbf{U}} = [\hat{u},\hat{v},\hat{\phi }]^\text {T}$ contains the spatially Fourier transformed prognostic variables, we have the following operators in the original equation form,

47 $$\begin{aligned} \mathcal {L} = \begin{bmatrix} 0 &  -1 &  i k\\ 1 &  0 &  0\\ i k &  0 &  0\\ \end{bmatrix}, \ \mathcal {N} = \mathcal {F} \left\{ - \begin{bmatrix} u\frac{\partial u}{\partial x} \\ u \frac{\partial v}{\partial x} \\ \frac{\partial }{\partial x} (u \phi ) \end{bmatrix} \right\} , \end{aligned}$$

where $\mathcal {F}\{\}$ denotes the spatial Fourier transform and *k* is the wavenumber. The eigenvalues of $\mathcal {L}$ identify the linear dispersion relations of $\omega = \pm \alpha \sqrt{1 + k^2 }$, with $\alpha \in \{ -1,0,1 \}$ denoting the dispersion relation branches. The fast modes of $\alpha = \pm 1$ correspond to inertia-gravity waves, and α=0 corresponds to Rossby waves, which are stationary on the *f*-plane due to the constant Coriolis parameter. Due to the zero frequency Rossby mode, the linear operator in this system is singular. The Moore-Penrose pseudoinverse of $\mathcal {L}^+$ and operator $\mathcal {L} \mathcal {L}^{+}$ are

48 $$\begin{aligned} \mathcal {L^+} = \begin{bmatrix} 0 &  \frac{1}{\psi ^2} &  \frac{-ik}{\psi ^2}\\ \frac{-1}{\psi ^2} &  0 &  0\\ \frac{-ik}{\psi ^2} &  0 &  0\\ \end{bmatrix}, \ \mathcal {L} \mathcal {L^+} = \begin{bmatrix} 1 &  0 &  0\\ 0 &  \frac{1}{\psi ^2} &  \frac{-ik}{\psi ^2}\\ 0 &  \frac{ik}{\psi ^2} &  \frac{k^2}{\psi ^2}\\ \end{bmatrix}, \end{aligned}$$

where $$\psi = \sqrt{1 + k^2}$$.

### Numerical implementation

We consider an initial condition at rest with a Gaussian perturbation of the geopotential height field,

49 $$\begin{aligned} u(x,0) = 0, \ \ \ v(x,0) = 0, \ \ \ \phi (x,0) = \exp \left( \frac{-(x-\pi )^2}{2} \right) - c_0, \end{aligned}$$

where $c_0$ is a constant chosen such $\int _0^{L_x} \phi ~\text {d}x = 0$ for a domain of length $L_x$. We solve the equations on the periodic interval $x \in [0,2 \pi ]$, which requires that

50 $$\begin{aligned} c_0 = \frac{1}{\sqrt{2\pi }} ~\text {erf} \left( \frac{\pi }{\sqrt{2}} \right) . \end{aligned}$$

We use a pseudospectral spatial discretisation with 32 grid points, which allows the matrix exponential to be expressed analytically in Fourier space as

51 $$\begin{aligned} e^{\pm \frac{t}{\epsilon } \mathcal {L}} = \begin{bmatrix} \cos (\frac{\psi t}{\epsilon }) &  \mp \frac{1}{\psi } \sin (\frac{\psi t}{\epsilon }) &  \pm \frac{ik}{\psi } \sin (\frac{\psi t}{\epsilon })\\ \pm \frac{1}{\psi } \sin (\frac{\psi t}{\epsilon }) &  \frac{1}{\psi ^2}(k^2 + \cos (\frac{\psi t}{\epsilon })) &  \frac{ik}{\psi ^2}(1-\cos (\frac{\psi t}{\epsilon }))\\ \pm \frac{ik}{\psi } \sin (\frac{\psi t}{\epsilon }) &  \frac{ik}{\psi ^2} (\cos (\frac{\psi t}{\epsilon }) -1) &  \frac{1}{\psi ^2} (1 + k^2 \cos (\frac{\psi t}{\epsilon }))\\ \end{bmatrix}. \end{aligned}$$

Alternate matrix exponential constructions for the RSWEs include rational approximations, e.g. [50, 51], or Chebyshev approximation [24].

Numerical dissipation is required to stabilise the pseudospectral method and is applied through a fourth-order hyperviscosity term $\mathcal {D}\textbf{U}$, where

52 $$\begin{aligned} \mathcal {D} = \begin{bmatrix} -\mu k^4 &  0 &  0\\ 0 &  -\mu k^4 &  0 \\ 0 &  0 &  -\mu k^4 \\ \end{bmatrix}. \end{aligned}$$

The diffusion coefficient is set to $\mu = 10^{-4}$. Although the diffusive term is linear, it is not included in $\mathcal {L}$. This is because including real eigenvalues in the linear operator leads to exponential growth and decay when performing phase-averaging for a timestepping equation (over phase variable *s*) or computing a local mean correction (over phase variable *r*); this means that the averages include amplitude variation and are no longer purely over the phase. We instead add the diffusive term to the right-hand side of the equations, which means replacing $\mathcal {N}$ with $\mathcal {N}^* (\textbf{U}) = \mathcal {N}(\textbf{U}) + \mathcal {D} \textbf{U}$ in the standard phase-averaging (6) and mean corrected phase-averaging (17) equations.

For the mean corrected method, the initial condition is computed with the fixed-point iteration of Algorithm 3 and $C_{\text {tol}} = 10^{-10}$. Using the local mean correction requires the selection of an (η, $\eta _C$) pairing; recall that η is the averaging window for phase-averaging of the timestepping equation, and $\eta _C$ is the averaging window for computing the local mean correction. We use the following procedure to choose a nominally best pair of windows, ($\eta ^*$, $\eta _C^*$):

1. Run simulations with the standard phase-averaged method over a range of normalised averaging windows $\zeta = \eta / \Delta t$ to find the window with the lowest error, $\eta ^*$. For the RSWEs, increments of $\Delta \zeta = 0.05$ are used.
2. Using the $\eta ^*$ value found in step 1, run simulations with the mean corrected method over a range of $\eta _C$ values to identify the local mean correction window with the lowest error, $\eta _C^*$. For the RSWEs, increments of $\Delta \eta _C = \epsilon$ are used.

We have chosen to use the same window of $\eta ^*$ for phase-averaging the timestepping equation with both methods; this means that the mean corrected method only needs to be examined over a range of $\eta _C$ values in step 2, instead of (η, $\eta _C$) combinations. This approach was found to be cheap and effective for initial studies, although it may miss the ($\eta ^*$, $\eta _C^*$) combination with the lowest error.

Simulation errors are computed as the sum of L2 errors in the *u*, *v*,  and ϕ fields, relative to a reference solution computed using the unaveraged modulation variable method (3), explicit RK4 timestepping, and $\Delta t = 10^{-3}$. We then compute a relative error reduction of the mean corrected method ($E_{\text {MC}}$) compared to standard phase-averaging ($E_{\text {PA}}$).

### Results

We investigate oscillation speeds of $\epsilon \in \{ 0.5, 0.1, 0.05, 0.01, 0.001 \}$ in both a shorter simulation of length $T_{\text {end}}=10$ and a longer simulation with $T_{\text {end}}=50$. Table 2 compares errors with the standard and locally mean corrected phase-averaging methods, along with the unaveraged modulation variable method ($E_{\text {MV}}$), for timesteps of $\Delta t \in \{0.1,0.2,0.3\}$. With an unaveraged modulation variable, the simulation errors grow as ϵ reduces. In all cases, standard phase-averaging is more accurate than the unaveraged modulation variable method, and this is especially pronounced for larger timescale separations.

For the instances with sufficiently fast oscillations (ϵ<0.5) the mean correction improves the accuracy for all timesteps and oscillation speeds. This indicates that the mean corrected algorithm performs at least as well as standard phase-averaging in these regimes, which is a known property of the classical mean correction [28]. Even better results with the mean correction may be obtained when using a η that differs from the best $\eta ^*$ from standard phase-averaging, i.e. finding the best ($\eta ^*$, $\eta _C^*$) combination within the full parameter space.

**Fig. 5:**
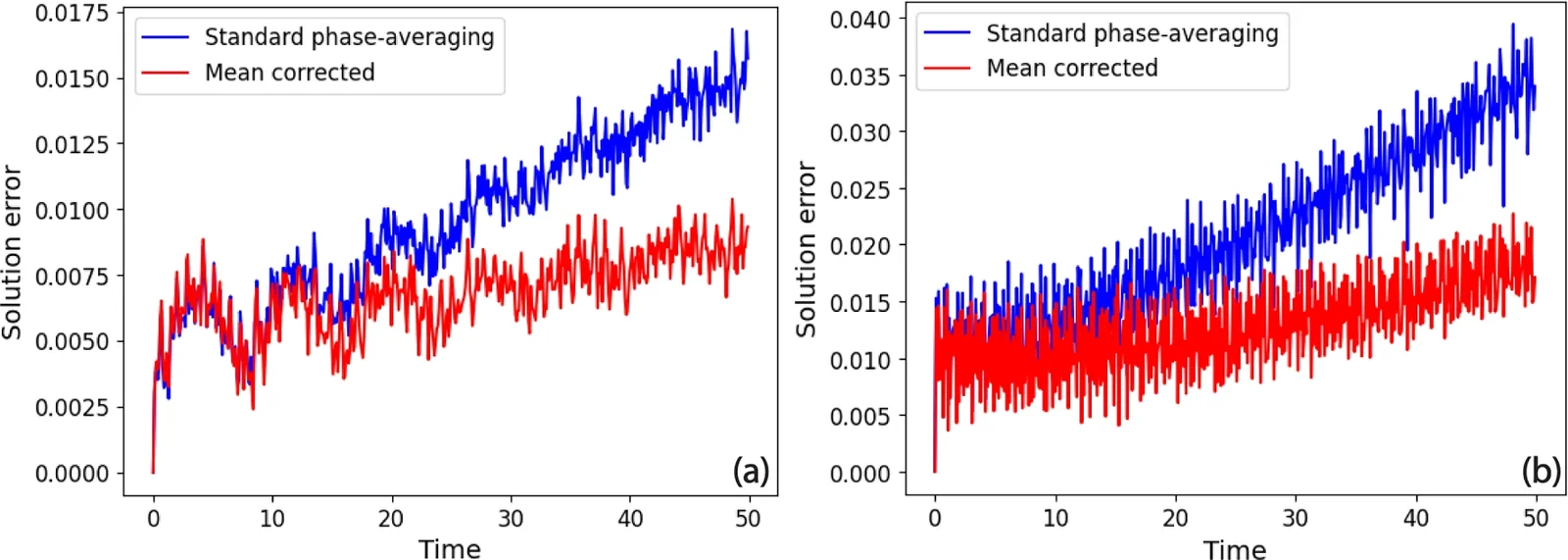
Errors over time from the standard and mean corrected phase-averaging methods in RSWEs experiments with Δt=0.1 and $T_{\text {end}}=50$, for ϵ=0.1 in (**a**) and ϵ=0.05 in (**b**). In these cases, the mean corrected method has a slower rate of error growth than standard phase-averaging, so differences in solution error become more pronounced further into the simulation

Improvements with the mean correction are largest for the ϵ=0.1 and ϵ=0.05 cases. Increasing the simulation length significantly improves the mean corrected performance for ϵ=0.1. An example of this is seen in Fig. 5a, with the greater accuracy improvement due to a slower error growth with the mean correction for t>20. Figure 5b shows similar behaviour with a faster oscillation speed of ϵ=0.05.

The mean correction improves phase-averaging accuracy for the fastest oscillation case of ϵ=0.001, even though the difference between the phase-averaged solutions is small. The reduced difference between the methods is due to the high accuracy of standard phase-averaging in this regime, with the modulation variable mapping capturing the majority of the key dynamics near the $\epsilon \rightarrow 0$ limit. This is the reason that operator splitting methods, which only retain zero frequencies in the modulation variable, are very accurate in rapidly oscillating regimes [8].

The mean corrected method, as currently formulated, struggles when ϵ=0.5. Using a larger timestep improves its performance, with Δt=0.3 leading to accuracy improvements with the mean correction, whilst the smaller timestep of Δt=0.1 leads to a degraded accuracy. A reason for the issues in this regime may be the assumption of $\delta \ll 1$ in deriving the mean corrected method (16), which we will return to in the discussion section.

**Table 2:** Comparing errors in the RSWEs simulations with the unaveraged modulation variable method ($E_{\text {MV}}$), standard phase-averaging ($E_{\text {PA}}$) and mean corrected phase-averaging ($E_{\text {MC}}$) for a range of oscillation speeds, timesteps, and short ($T_{\text {end}}=10$) and long ($T_{\text {end}}=50$) end times

		ϵ=0.5				ϵ=0.1			
Δt	$T_{\text {end}}$	$\phantom {\displaystyle \frac{1}{2}}E_{\text {MV}}$	$E_{\text {PA}}$	$E_{\text {MC}}$	δE	$E_{\text {MV}}$	$E_{\text {PA}}$	$E_{\text {MC}}$	δE
0.1	10	0.00029	0.00028	0.00205	-	0.0084	0.0051	0.0046	9.3%
0.2	10	0.0068	0.0056	0.0055	1.1%	0.0490	0.0181	0.0155	14.5%
0.3	10	0.0207	0.0114	0.0101	11.7%	0.1661	0.0295	0.0249	15.6%
0.1	50	0.00052	0.00037	0.00285	-	0.0126	0.0096	0.0068	29.1%
0.2	50	0.0072	0.0058	0.0066	-13.6%	0.0929	0.0425	0.0317	25.6%
0.3	50	0.0249	0.0184	0.0183	0.4%	0.2719	0.0454	0.0348	23.4%
		ϵ=0.05				ϵ=0.01			
Δt	$T_{\text {end}}$	$E_{\text {MV}}$	$E_{\text {PA}}$	$E_{\text {MC}}$	δE	$E_{\text {MV}}$	$E_{\text {PA}}$	$E_{\text {MC}}$	δE
0.1	10	0.0260	0.0100	0.0080	19.1%	0.0537	0.0061	0.0061	1.0%
0.2	10	0.0930	0.0117	0.0096	17.6%	0.0182	0.0078	0.0078	0.0%
0.3	10	0.1301	0.0228	0.0204	10.4%	0.2020	0.0089	0.0083	6.8%
0.1	50	0.0499	0.0207	0.0129	38.0%	0.0969	0.0161	0.0145	9.9%
0.2	50	0.1652	0.0163	0.0145	10.6%	0.4203	0.0203	0.0171	15.7%
0.3	50	0.1872	0.0363	0.0331	8.6%	0.3294	0.0168	0.0157	6.4%
		ϵ=0.001							
Δt	$T_{\text {end}}$	$E_{\text {MV}}$	$E_{\text {PA}}$	$E_{\text {MC}}$	δE				
0.1	10	0.06714	0.00093	0.00089	5.3%				
0.2	10	0.03694	0.00084	0.00077	8.0%				
0.3	10	0.15338	0.00065	0.00062	3.5%				
0.1	50	0.09932	0.00203	0.00199	2.0%				
0.2	50	0.74501	0.00203	0.00199	2.0%				
0.3	50	0.31249	0.00191	0.00178	7.0%				

The percentage relative error improvement of mean corrected phase-averaging compared to standard phase-averaging is quoted as δE. Instances for ϵ=0.5 where the mean corrected method performs poorly are denoted with a dash. In all cases, standard phase-averaging leads to a lower error than the unaveraged modulation variable method. For ϵ<0.5, the mean correction leads to smaller errors than standard phase-averaging for all cases

**Fig. 6:**
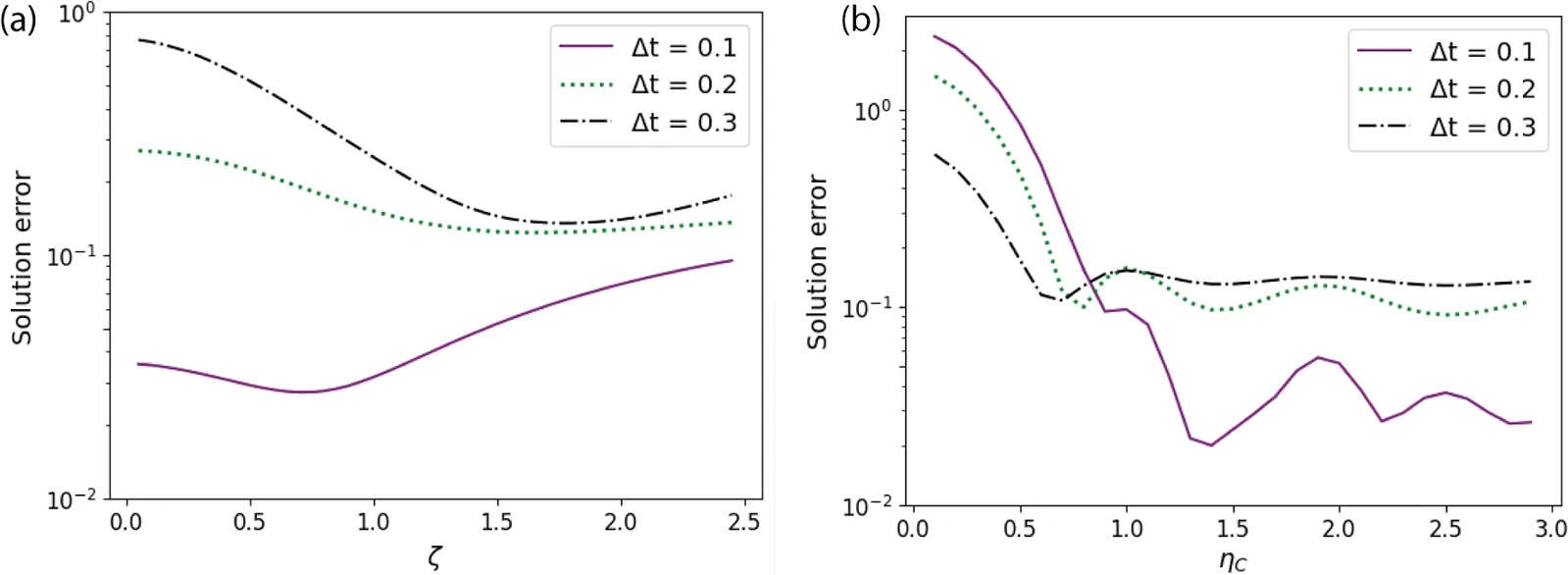
Peddle plots for the longer $T_{\text {end}}=50$ simulations with ϵ=0.1. Standard phase-averaging errors for different normalised averaging windows are shown in (**a**). As expected, there is a minimum in the error for each timestep size. Mean corrected errors are shown in (**b**) for different $\eta _C$, when using the corresponding $\eta ^*$ from (**a**). There are now three local minima, with the best choice depending on the timestep size: $\eta _C^* = 1.4,2.5,0.7$ for Δt=0.1,0.2,0.3, respectively. Values of $\eta _C^* = 1.4,2.5$ are also best for the shorter ϵ=0.1 simulations with $T_{\text {end}}=10$ (Table 3)

Figure 6 provides insight into the selection of the $\eta ^*$ and $\eta _C^*$ windows for the standard phase-averaging and the mean corrected methods, using the longer simulation time and ϵ=0.1. Figure 6a shows a Peddle plot for standard phase-averaging, with a clear minimum for each timestep size, although at different values of $\zeta = \eta /\Delta t$. The Peddle plot for the local mean correction in Fig. 6b shows mean corrected errors over $\eta _C$, using the $\eta ^*$ value identified in Fig. 6a. Now, there are multiple minima, showing that there is not a single window size that is clearly best for computing the local mean correction. The presence of minima, instead of a monotonic decay in error with $\eta _C$, shows the advantage of retaining low-frequency components in the local mean correction (13), instead of using the classical mean correction of $\eta _C \rightarrow \infty$ (12). In Fig. 6b, each timestep size leads to roughly three local minima in the range of windows examined, with the best window size varying with Δt. One possibility for these multiple local minima is periodicity in the matrix exponential (51) appearing in the local mean measure of nonlinear oscillation.

Table 3 investigates the link between the linear oscillation speed, ϵ, and the selection of the best averaging windows. The majority of the best phase-averaging windows lie in the interval $\zeta \in [0.5,1.5]$, which is consistent with the findings of [41] that the best averaging window is proportional to the timestep size. For the local mean correction, the best averaging window of $\eta _C^*$ depends on the oscillation speed. Fewer linear oscillations (*N*) are contained within $\eta _C^*$ with reduced ϵ (Table 3). This makes the local mean correction cheaper to compute in the faster oscillation regimes, with fewer points ($K_r$) required in the local mean correction averaging kernel ($\mathcal {K}_C$). This contrasts the increasing computation required for phase-averaging the timestepping equation ($K_s$) with a smaller ϵ (34), as more frequent sampling is required for faster oscillation speeds.

**Table 3:** The best averaging window sizes identified in the RSWEs experiments with the shorter simulation time of $T_{\text {end}}=10$

	ϵ=0.5			ϵ=0.1			ϵ=0.05		
Δt	$\zeta ^*$	$\eta _C^*$	*N*	$\zeta ^*$	$\eta _C^*$	*N*	$\zeta ^*$	$\eta _C^*$	*N*
0.1	0.2	28.5	57	0.95	1.4	14	1.35	0.75	15
0.15	0.45	29	58	1.1	1.4	14	1.4	0.75	15
0.2	0.85	23	46	1.25	1.4	14	1.25	0.4	8
0.25	0.95	23	46	1.4	2.5	25	1.5	0.2	4
0.3	1.0	11	22	1.4	2.5	25	1.45	0.25	5
	ϵ=0.01			ϵ=0.001					
Δt	$\zeta ^*$	$\eta _C^*$	*N*	$\zeta ^*$	$\eta _C^*$	*N*			
0.1	1.35	0.08	8	0.55	0.004	4			
0.15	1.45	0.04	4	0.4	0.002	2			
0.2	1.3	0.07	7	0.3	0.004	4			
0.25	1.35	0.04	4	0.35	0.004	4			
0.3	1.35	0.04	4	0.2	0.002	2			

The best normalised averaging window of $\zeta ^* = \eta ^*/\Delta t$ is found with the standard phase-averaging method. The best window for the local mean correction is $\eta ^*_C$, which uses the corresponding $\zeta ^*$ value. $N = \eta _C^*/\epsilon$ shows the number of linear oscillations that are contained within $\eta _C^*$. Smaller values of *N* are favoured as ϵ gets smaller, which reduces the computation needed for the local mean correction

**Fig. 7:**
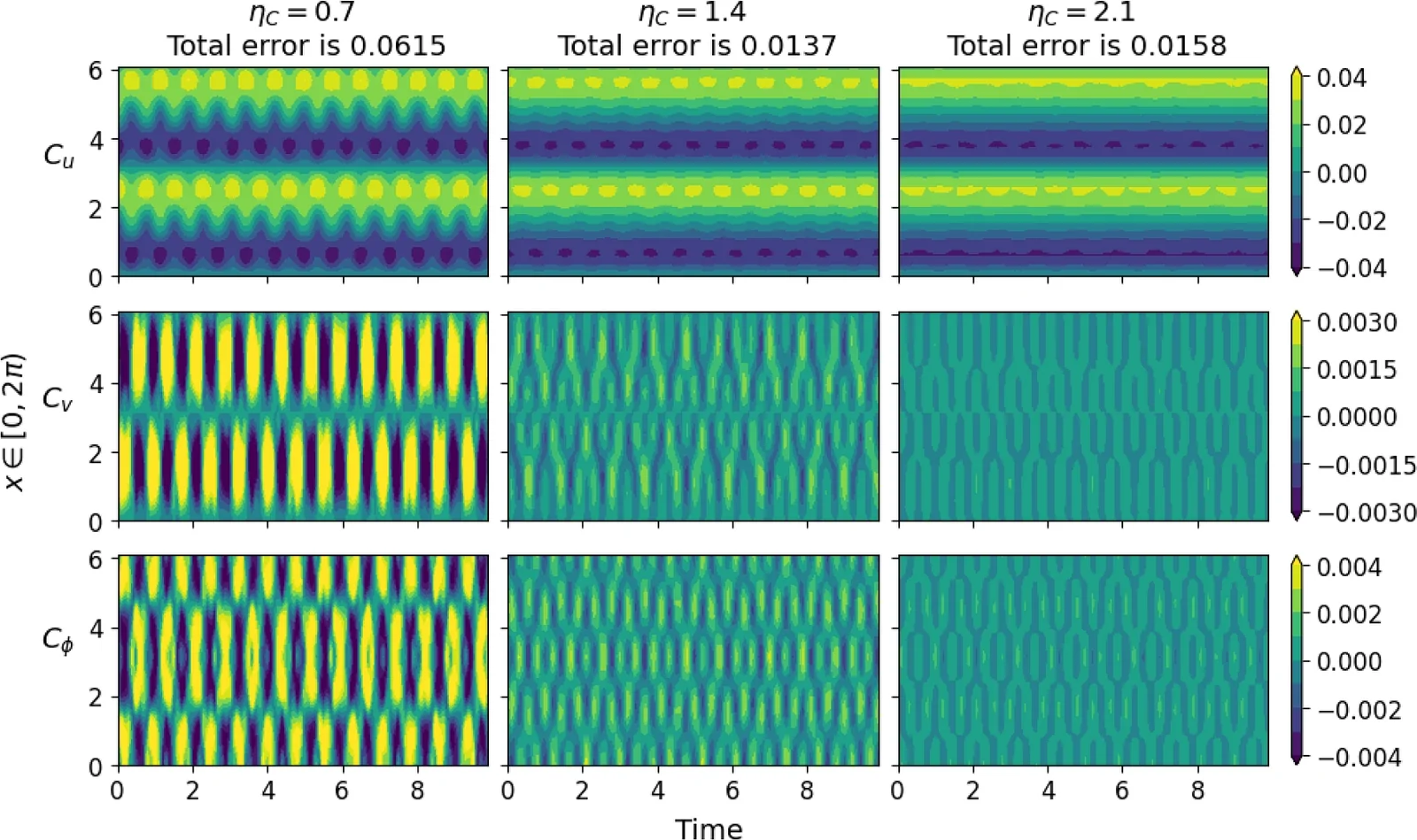
Visualising the local mean correction in the RSWEs experiment with $\epsilon =0.1, \Delta t =0.1, T_{\text {end}}=10$. These physical space visualisations are obtained from the inverse Fourier transform of the Fourier space mean correction. Striking differences in the mean correction are observed depending on the value of the mean correction averaging window, $\eta _C$. The underaveraged case ($\eta _C=0.7$) has the largest corrections and the most variation in $C_u$ over time, whilst the largest window ($\eta _C=2.1$) has the smallest magnitudes and the greatest homogenisation of $C_u$. The best window of $\eta _C=1.4$ is a balance of a sufficient time interval to accurately compute a mean, yet retaining local variation depending on the current solution value. The magnitude of $C_u$ is larger than $C_v$ and $C_{\phi }$ in these experiments. We can see visually that δ (15), which is proportional to the time derivative of $\textbf{C}$, reduces with larger $\eta _C$

Lastly, Fig. 7 visualises the impact of $\eta _C$ on the local mean correction of $\textbf{C}=[C_u, C_v, C_{\phi }]$. Three values of $\eta _C$ are compared in the $\epsilon =0.1, ~\Delta t =0.1, ~T_{\text {end}}=10$ case: the best window of $\eta _C = 1.4$, a smaller window of $\eta _C=0.7$ that leads to a much larger error, and $\eta _C=2.1$, which is slightly off the best accuracy. With the Gaussian initial condition (49), the $C_u$ correction term is an order of magnitude larger than $C_v$ or $C_{\phi }$. The correction components have two periods in the spatial domain, due to each term in $\mathcal {N}$ being quadratic. The local mean correction has striking visual differences depending on the averaging window size and the spectrum of frequencies that this retains. The smallest window of $\eta _C=0.7$ leads to the largest mean corrections, as there is less cancellation of oscillations. Conversely, the mean corrections for $\eta _C=2.1$ are the smallest in magnitude, and $C_u$ is more homogenised in time as more non-zero frequency interactions are removed. With $\eta _C=1.4$, $C_u$ balances the retention of local information, through higher frequency components, against a smoother time evolution; this leads to the most accurate mean correction in this instance.

## Discussion

This paper introduced a mean corrected version of phase-averaging for improved timestepping accuracy in oscillatory, geophysical PDEs. We presented algorithms for a local mean correction and the mean corrected phase-averaging method, which extends the finite phase-averaging framework of [20]. In the swinging spring ODE tests, the mean correction reduced phase errors within the modulation variable space and was effective for a range of resonance factors that mimic direct, near, and far resonance wave interactions in the RSWEs. We then saw accuracy improvements with a local mean correction in the one-dimensional RSWEs, which shows the potential for mean corrected phase-averaging in idealised geophysical fluid flow applications.

The local mean correction is a new quantity that combines the idea of a mean correction [28] with the benefits of a finite phase-average [41]. The retention of low-frequency information in the local mean correction is beneficial when using a finite phase-average of the timestepping equation. The best window length for the local mean correction, $\eta _C^*$, balances a sufficiently long interval to compute a mean value with a small enough interval that retains pertinent local variation. This trade-off is similar to the best window of $\eta ^*$ for phase-averaging of the timestepping equation, which balances stiffness reduction from large timesteps with retaining important low-frequency dynamics that are removed with a classical time-average. Beyond improving phase-averaging accuracy, the local mean correction may represent an important physical quantity for the long-time solution of geophysical fluid systems, and is worth further exploration.

There are several immediate ways to improve the mean corrected phase-averaging algorithm for greater accuracy and computational efficiency. First, visualisations of the mean correction in the RSWEs (Fig. 7) indicate that, though $\textbf{C}$ may be small, its rate of change could be better approximated. Therefore, one possibility for advancing the algorithm is avoiding the assumption that $\delta \ll 1$ (15) and instead taking $\delta = \delta _{\Delta t}$, where $\delta _{\Delta t}$ is constant over one timestep. This adds a term of the form $\epsilon \delta _{\Delta t}$ into the mean corrected timestepping Eq. (16), with the potential for further phase-averaging error reduction. Second, a faster algorithm can be constructed using precomputed matrix exponentials of $\exp (\pm \Delta t \mathcal {L}/\epsilon )$ rather than evaluating $\exp (\pm t \mathcal {L}/\epsilon )$ at each timestep. Examples of this approach with phase-averaging are the Strang-split method of [12] and the Lawson-RK approach, introduced for phase-averaging in [24] and adapted for mean corrected phase-averaging in Chapter 7 of [52]. Third is the more effective selection of $\eta ^*$ and $\eta _C^*$, such as through an optimisation algorithm, or selections of time-dependent $\eta ^*(t)$ and $\eta _C^*(t)$ during the simulation instead of selections in advance. Fourth, allowing the mean corrected method to use a different $\eta ^*$ from standard phase-averaging could allow further accuracy improvements.

To better evaluate the strengths and weaknesses of the new algorithm, it would be beneficial to perform experiments in other geophysically relevant differential equations. One related system is the Klein-Gordon-type PDE, which contains the same linear dispersion relation as the inertia-gravity waves of the RSWEs but without the slow mode; [52] previously showed phase-averaging improvements with a local mean correction in this system. Another useful test would be the Boussinesq equations, which were investigated with phase-averaging in [53]. We also wish to include the full Coriolis force in the RSWEs, which means that the slow waves have a nonzero phase speed. The generalisability of the algorithm is also worth exploring, such as its performance when the matrix exponential uses a linearisation about a mean flow. Lastly, we wish to test the new algorithm for parallel-in-time applications, primarily as a modified coarse propagator in the oscillatory parareal method of [20]. As the local mean correction can be computed in parallel, it could provide a cheap modification to the coarse propagator to improve accuracy.

## Data Availability

Python scripts for running the numerical experiments can be accessed at the GitHub repository: https://github.com/ta440/mean_corrected_phase_averaging.

## Appendix A: cancellation of oscillations for the swinging spring mean correction

Consider the first term in the *x* component of $\mathcal {N}(e^{-t \mathcal {L} }\textbf{W})$ in the modulation variable swinging spring system (42),

A1 $$\begin{aligned} \frac{i \lambda }{4 \omega _R} A \tilde{x} \tilde{z} = \frac{i \lambda }{4 \omega _R} e^{-i (\rho + 1) \omega _R t} \tilde{x}(t) \tilde{z}(t). \end{aligned}$$

Compute the mean contribution of this term, $C_{Axz}$, by applying (12),

A2 $$\begin{aligned} C_{Axz} = \lim _{\eta _C \rightarrow \infty } \frac{1}{\eta _C} \int _{r=-\eta _C/2}^{\eta _C/2} \frac{i \lambda }{4 \omega _R} e^{-i (\rho + 1) \omega _R (t + r)} \tilde{x}(t) \tilde{z}(t) \text {d}{r}. \end{aligned}$$

The averaging is performed over oscillations at a fixed solution value, allowing most terms to be taken outside the integral. This leads to

A3 $$\begin{aligned} C_{Axz} = \frac{i \lambda }{4 \omega _R} e^{-i (\rho + 1) \omega _R t} \tilde{x}(t) \tilde{z}(t) \lim _{\eta _C \rightarrow \infty } \frac{1}{\eta _C} \int _{r=-\eta _C/2}^{\eta _C/2} e^{-i (\rho + 1) \omega _R r} \text {d}{r} = 0, \end{aligned}$$

where we have used the following property of complex exponentials:

A4 $$\begin{aligned} \lim _{\eta _C \rightarrow \infty } \frac{1}{\eta _C} \int _0^{\eta _C} e^{i \phi t} \text {d}t = {\left\{ \begin{array}{ll} 0, ~\text {if} \ \phi \ne 0, \\ 1, ~\text {if} \ \phi = 0. \end{array}\right. } \end{aligned}$$

Accordingly, every term in the modulation variable swinging spring system with a complex exponential component (43) will have this cancellation of oscillations for $\rho \in [1.5,2.5]$, resulting in a zero mean value and no contribution to $\textbf{C}_{\infty }$.
